# Effectiveness of e-share intervention for caregivers of elderly people after stroke: a pragmatic randomized trial*

**DOI:** 10.1590/1518-8345.7414.4467

**Published:** 2025-02-17

**Authors:** Francine Melo da Costa, Débora Francisco do Canto, Lisiane Manganelli Girardi Paskulin

**Affiliations:** 1 Universidade Federal do Rio Grande do Sul, Escola de Enfermagem, Porto Alegre, RS, Brazil; 2 Hospital de Clínicas de Porto Alegre, Serviço de Enfermagem Ambulatorial, Porto Alegre, RS, Brazil; 3 Hospital de Clínicas de Porto Alegre, Serviço de Enfermagem Clínica, Porto Alegre, RS, Brazil; 4 Hospital de Clínicas de Porto Alegre, Diretoria de Enfermagem, Porto Alegre, RS, Brazil; 5 Scholarship holder at the Conselho Nacional de Desenvolvimento Científico e Tecnológico (CNPq), Brazil

**Keywords:** Caregivers, Education, Stroke, Hospital to Home Transition, Aged, Educational Technology

## Abstract

**Objective:**

to analyse the effectiveness of a virtual educational intervention for family caregivers on the burden and ability to care for elderly people after stroke.

**Method:**

randomized pragmatic trial with 58 caregivers of elderly survivors of stroke. The intervention group received access to an online course for caregiver education. Caregiver training was assessed using the Scale of Capabilities of Informal Caregivers for Dependent Elderly People due to Stroke and burden through the Caregiver Burden Scale before and three months after hospital discharge, both adapted and validated for use in Brazil. The Generalized Estimating Equations model complemented by the Least Significant Difference was adopted.

**Results:**

the sample was homogeneous and the groups differed statistically only in relation to kinship (p=0.034), with a higher proportion of children observed in the control group. There was an effect of the intervention on improving medication administration (p=0.006) and reducing disappointment (p=0.011) in the intervention group.

**Conclusion:**

the intervention benefited the caregivers who received it in terms of improving medication administration and reducing disappointment. This is the first study in Brazil that proposes the use of digital educational technologies for this group, representing an important advance in Nursing. Clinicaltrial.gov registration: NCT05553340.

## Highlights

**(1)**First study in Brazil that proposes a MOOC for caregivers of elderly people after stroke. **(2)**Advances in Nursing in the construction of digital educational technologies. **(3)**E-share helped caregivers improve medication administration. **(4)**E-share helped caregivers reduce overload in the disappointment domain. **(5)**Digital tools contribute to the use of nurses’ work.

## Introduction

Stroke is a neurological condition that mainly affects the elderly, and is the second leading cause of death in Brazil and worldwide^(1)^. In Brazil, between January and October 2023, approximately 150,000 people were hospitalized due to stroke, of which approximately 117,000 (79%) were 60 years old or older, and around 18,000 (28%) died^(2)^. In addition to the high mortality rate, stroke is considered one of the main causes of disability in the elderly population. Elderly people who suffer a stroke have more limitations in self-care after hospital discharge, requiring assistance^(1)^.

Informal care involves numerous tasks, such as practical, emotional, financial, and social support^(3)^. In Brazil, the availability of support from formal networks is limited, with the family being responsible for most of the care^(4)^. Furthermore, support for informal caregivers is not explicitly provided for in public policies^(5)^, leaving families with the responsibility of providing care, often without the necessary information, qualifications and resources^(6)^.

Specifically, regarding informal caregivers of dependent elderly people who have suffered a stroke, a study identified that the main care activities performed were: providing materials and/or support for feeding, dressing and taking care of medications. The activities in which caregivers presented the greatest difficulty were transferring and positioning^(7)^.

The aforementioned studies reveal the existence of gaps in the quality of care provided to families. In contrast, international recommendations for the transition from hospital to home care indicate that the education of family caregivers should include training in personal care techniques, such as feeding, transferring and positioning^(8)^.

International studies have also shown that educational interventions aimed at family caregivers of elderly individuals after a stroke help reduce burden^(9)^, improve the ability to assist the elderly at home^(9)^, improve the caregiver’s Quality of Life (QoL)^(8)^ and have a positive impact on the physical and mental health of survivors^(8)^.

Therefore, there is still no evidence, in the national context and in conditions closer to those found in care practice, that an educational intervention based on the development of skills and knowledge can improve the ability of caregivers to provide care to the elderly. Furthermore, virtual interventions aimed at caregivers are recent alternatives that deserve to be further tested^(10)^.

The present investigation was developed considering the hypothesis that virtual educational intervention, carried out by nurses for family caregivers of elderly individuals after a stroke, improves the ability to provide care, compared to usual monitoring. The concept of “capacity” adopted in this study comes from Social Cognitive Theory, in which the author translates knowledge, skills and competencies as a result of the term “capacity”^(11-12)^.

This study explores alternatives for transitioning care, optimizing resources, improving the care experience and making the best use of nurses’ working time, as well as highlighting and strengthening their educational role. The aim was to analyse the effectiveness of a virtual educational intervention for family caregivers, in terms of the burden and ability to care for elderly people after a stroke, when compared with usual care guidelines, in the three-month period after hospital discharge.

## Method

### Design

Randomized Pragmatic Study (RPS), called Educational Intervention With Digital Technology For Family Caregivers (e-share), registered at clinicaltrial.gov under number NCT05553340. The research followed the recommendations of the Consolidated Standards of Reporting Trials (CONSORT) extension^(13)^.

### Setting and period

Study conducted in the Emergency Department, in five clinical inpatient units and in the Stroke Special Care Unit (SCU-SCU) of a large university hospital in southern Brazil.

The SCU-SCU treats patients diagnosed with stroke, has 10 beds and offers monitoring by a multidisciplinary team composed of doctors, nurses, pharmacists, nutritionists, physiotherapists, speech therapists, social workers and psychologists. Upon discharge, these professionals provide patients and their families with information about the disease, as well as educational materials regarding the care that will be provided at home. Depending on the capacity of these beds or the severity of the patient’s condition, they may be allocated to other inpatient units or discharged directly from the emergency room^(14)^.

Recruitment took place from January to June 2023, and data collection took place from January to November 2023.

### Population and sample

The study participants were family caregivers of elderly individuals aged 60 or over. A family caregiver is understood as the person responsible for providing unpaid care to the elderly at home, and who may or may not be a family member^(15)^.

Family caregivers over the age of 18 who were the main unpaid caregivers for patients aged 60 or over (of both genders) with a medical diagnosis of stroke during their current hospitalization were included. Family caregivers who: (a) did not have access to the internet; (b) were not able to access the virtual intervention, as verified using the checklist for access and navigation to the course, developed for this study; (c) did not have a telephone line for contact; (d) were accompanying elderly individuals who were transferred to long-term care facilities after discharge; (e) were accompanying elderly individuals who died during the participant recruitment phase were excluded of the sample.

The sample size to detect differences in the mean of the scale used to assess caregivers’ training between the intervention and control groups was calculated using the Power and Sample Size for Health Researchers (PSS Health) tool^(16)^. A power of 80%, a significance level of 5%, and a Cohen’s effect size of 0.8 were considered. The higher the Cohen’s d value, the more significant the discrepancy between the means; d values of 0.8 represent large effects^(17)^. This was adopted since, at the time of this research, there were no previous studies that applied the adopted scale, seeking to identify differences in the means.

The estimated total sample size reached 52 participants, 26 in each group. Adding 10% for possible refusals, the sample size was 58 participants, 29 in each group. Eligible participants were family caregivers of patients aged 60 or over, with a medical diagnosis of stroke, admitted to the institution’s units, monitored by electronic medical records available in the institutional software, during the data collection period.

### Variables and instruments

The primary outcome was the ability of informal caregivers to care for elderly individuals after stroke, and the secondary outcome was caregiver burden. Participants were assessed at baseline, during hospital admission, and at the final assessment, 90 days after discharge.

Sociodemographic and clinical information were collected from the elderly individuals and their family caregivers. The Functional Independence Measure (FIM) was also assessed for stroke survivors. The FIM quantitatively assesses the burden of care that a dependent patient demands from another person when performing motor and cognitive tasks. It consists of 10 categories divided into 6 dimensions: self-care; sphincter control; transfers; locomotion; communication; and social cognition. Overall, FIM scores can range from 18 to 126. The lower the score, the greater the elderly individual’s dependence to perform the tasks. The scale underwent a validation process for the Brazilian context in 2004, presenting good correlation in the test/retest and in interobserver reproducibility (Pearson: 0.91-0.98; intraclass correlation coefficient – ICC: 0.91-0.98 and Pearson: 0.87-0.98; ICC: 0.87-0.98, respectively)^(18)^.

The primary outcome was verified through the Scale of Capabilities of the Informal Caregiver for Dependent Elderly People due to Stroke (CICDEP-STROKE), which assesses the different capabilities that family caregivers have, or need to improve, in providing care to dependent elderly people after a stroke. Adapted and validated for use in Brazil, the CICDEP-STROKE has satisfactory test-retest reliability (ICC = 0.94; 95% confidence interval = 0.91-0.96) and excellent internal consistency reliability (Cronbach’s alpha = 0.914)^(19)^.

The scale consists of 29 items that address issues related to the following activities: eating/drinking (orally or via nasogastric tube/gastrostomy); medication administration; hygiene care (bathing and elimination); skin care; dressing/undressing; and transferring/positioning. These items are evaluated on a scale from 0 (does not demonstrate - does not perform the activity) to 3 (fully demonstrates - performs the activity correctly and independently). In addition, the response option “NA – not applicable” can be used in cases where the caregiver does not perform a certain activity because the elderly person does not require care^(19)^. The total score on the scale ranges from 0 to 87 points, with no cutoff point, and the higher the score, the more capable the caregiver is^(19)^.

The secondary outcome was assessed using the Caregiver Burden Scale (CBS)^(20)^ , which has 22 items divided into 5 dimensions: general tension, isolation, disappointment, emotional involvement, and environment. The questions can be answered from 1 - not at all to 4 - often. The total score is obtained by the arithmetic mean of the values equivalent to the answers to the 22 questions, and the individual score is obtained from the mean of the values equivalent to the answers to the questions in each dimension. There is no cutoff point; the higher the score, the greater the burden^(20)^. The scale was adapted and validated for the Brazilian context, and the intra- and inter-observer reproducibility coefficients were 0.87 and 0.92, respectively^(20)^.

### Data collection

Data collection began with the identification of patients aged 60 or over, with a medical diagnosis of stroke upon admission, through daily consultation of the hospital’s computerized system. The elderly individuals selected received a visit from the evaluating researchers to verify the existence of a family caregiver and whether he or she and the elderly individual met the inclusion criteria for the study, and to apply the eligibility checklist for access to the course. If the caregiver present at that time was not the primary caregiver, a telephone contact was made to verify whether the inclusion criteria were met, and the first assessment was scheduled. Those who agreed to participate signed the Free and Informed Consent Form (FICF) and, subsequently, the baseline data were collected.

The caregiver was contacted by telephone, three months after discharge (90 days), for the final assessment, by the same researchers responsible for the initial assessment. The collection was carried out via online conference via Google Meet® or video call via telephone. The three-month interval complied with the recommendations of the Brazilian reproducibility study of the CICDEP-STROKE scale^(19)^, and in accordance with evidence regarding the effectiveness of implementing educational intervention programs that suggest at least a three-month interval between assessments^(8)^.

The inter-rater agreement of the scales under study was assessed before and after training for data collection. An ICC greater than 0.70 and a significance level of 5% (p<0.05) were adopted, and the analyses were performed using the Statistical Package for the Social Sciences (SPSS) program, version 27.0. After training, all ICCs were greater than 0.70 and statistical significance was found (p<0.05).

### Randomization and blinding

After the instruments were applied, participants were randomized to a Control group (CG) or Intervention group (IG). To do this, a list generated by the website https://www.randomizer.org/ was used, which follows a numerical order in which each number is already randomly assigned to one of the groups. The evaluators contacted a professional outside the research group responsible for the allocation.

The researchers who performed the initial and final assessment and the recruitment of participants were blinded to the allocation. During the intervention, the family caregiver was informed that they would receive the final assessment and was instructed not to mention that they had received the educational intervention. Likewise, the CG was instructed, via telephone contact by a member outside the research group, not to inform the evaluator that they had not received the intervention. The interventionist researchers only met the participants in the IG.

### Intervention

The multicomponent intervention aimed to equip family caregivers to assist the elderly in activities of daily living (ADLs) after discharge. The intervention was carried out by two nurses - interventionist researchers, through a massive open online course (MOOC). Details on the design, construction and content of the MOOC can be found in a previously published article^(21)^.

In addition to making the MOOC available, the intervention included telephone contact with participants 7, 30, 60 and 80 days after hospital discharge to check the progress of the course and resolve any difficulties. A hotline was also made available to participants, from Monday to Friday, from 8:00 am to 6:00 pm, answered by nurses, in case there were any questions regarding access and navigation of the course. The intervention lasted three months.

After allocation, those included in the IG received an instructional visit by the interventionist researchers, while still in hospital, with the aim of providing detailed guidance on the intervention. In addition, caregivers were given guidance on telephone contacts and the availability of the hotline. At this time, a pamphlet containing instructions was handed out, which was also sent via *WhatsApp* ^®^.

### Control

For the control group, the intervention was not offered. The patient and his/her family caregiver received usual care guidelines and conventional monitoring in the service network to which they have access (public or private), as did the participants in the IG.

### Data analysis

The intention-to-treat technique was adopted^(17)^. To compare means, the Student’s t-test was applied. In case of asymmetry, the Mann-Whitney test was used. To compare proportions, the Pearson chi-square or Fisher’s exact tests were applied.

To compare the intergroup and intragroup scales simultaneously, the Generalized Estimating Equations (GEE) model complemented by the Least Significant Difference (LSD) test was used. The linear model was applied to the numerical variables with normal distribution and the logarithmic model to those with asymmetric distribution. In the same model, the difference in kinship between the two groups and the total number of accesses and modules accessed for the IG were adjusted. The significance level adopted was 5% (p<0.05), and the analyses were performed in the SPSS program, version 27.0.

### Ethical aspects

The rules of Resolution No. 466/2012 of the National Health Council^(22)^ and Circular Letter No. 2/2021/CONEP/SECNS/MS on research in a virtual environment^(23)^ were observed. The participants signed an informed consent form. The research was approved by the Research Ethics Committee, Certificate of Presentation of Ethical Appreciation (CAAE): 59589922.0.0000.5327, and the research protocol was registered at clinicaltrial.gov under identification NCT05553340.

## Results

**Figure 1 -:**
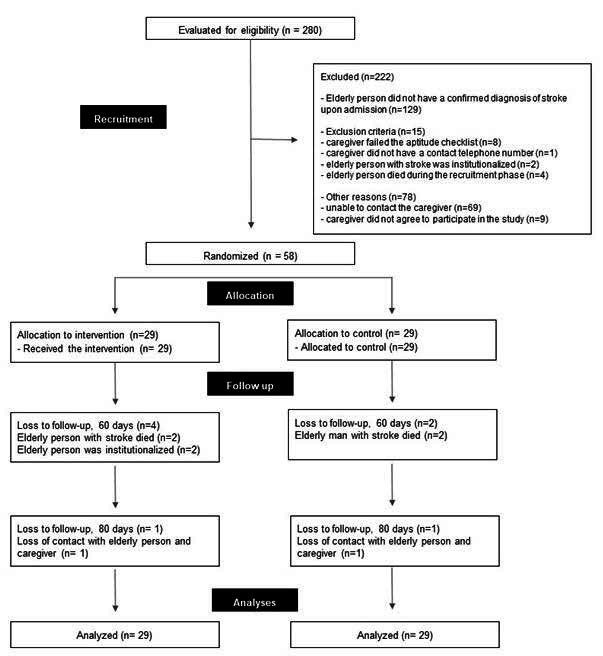
Study diagram according to Consolidated Standards of Reporting Trials (CONSORT)

During the recruitment period, 280 caregivers were assessed for eligibility, of which 222 were excluded: 129 for not meeting the inclusion criterion “caring for elderly individuals with a confirmed diagnosis of stroke during the current hospitalization”; 15 for meeting at least one exclusion criterion; and 78 for other reasons: a) nine for not accepting to participate in the study, and b) 69 for not being able to contact the caregiver after three attempts on different days and shifts. The sample consisted of 58 participants, 29 of whom were allocated to the IG and 29 to the CG. The study diagram according to CONSORT is presented in Figure 1.

The sociodemographic characteristics, health status and caregiver status in the IG and CG are presented in Table 1. The groups differed statistically only in relation to kinship (p=0.034), with a higher proportion of children observed in the CG. The sociodemographic characteristics and health status of the elderly stroke survivors were similar between the groups and are presented in Table 2. It is noteworthy that although there was no significant difference between the groups in relation to the FIM, the total score increased in both groups when comparing the baseline and final assessments, with this intragroup difference being statistically significant (p<0.001).

**Table 1 -:** Sociodemographic characteristics, health status and caregiver status of family caregivers (n = 58). Porto Alegre, RS, Brazil, 2024

**Variables**	IG* **(n=29)**	**CG** ^†^ **(n=29)**	**p**
Age (years)^‡^	52.1 ± 13.3	51.4 ± 11.6	0.818
Gender - Female^§^	22 (75.9)	24 (82.8)	0.746
Marital status^§^- With a partner	18 (62.1)	14 (48.3)	0.428
Professional Status^§^- With occupation	15 (51.7)	20 (69.0)	0.283
Education^§^			0.840
Elementary school	5 (17.2)	6 (20.7)	
High School	14 (48.3)	15 (51.7)	
Higher education/Post-graduation studies	10 (34.5)	8(27.6)	
Kinship^§^			0.034
Son	13 (44.8)	18 (62.1)	
Spouse	6 (20.7)	9 (31.0)	
Other Family members	10 (34.5)	2 (6.9)	
Health problems^§^	15 (51.7)	18 (62.1)	0.596
Cardiovascular diseases	9 (31.0)	14 (48.3)	0.283
Diabetes Mellitus	2 (6.9)	3 (10.3)	1.000
Family income^||^(minimum wage)^¶^	3000 (2500-5000)	2640 (1950-3950)	0.180
Lives with an older person (yes)^§^	20 (69.0)	18 (62.1)	0.550
Time living with the older person (years)^||^	20 (5 – 45)	30 (10 – 43)	0.361
Weekly care days^‡^	6.0 ± 1.7	6.1 ± 1.8	0.878
Experience as a caregiver (yes)^§^	20 (69.0)	22 (75.9)	0.769
Receive help with care (yes)^§^	17 (58.6)	12 (41.4)	0.294
Types of help^§^			
Instrumental	16 (94.1)	8 (66.7)	0.130
Emotional	6 (35.3)	5 (41,7)	1,000
Financial	10 (58,8)	4 (33,3)	0,329
Has received training to care for someone^§^	1 (3,4)	6 (20,7)	0,102

*IG = Intervention group; ^†^CG = Control group; ^‡^Mean ± standard deviation; ^§^Absolute number and (%); ^||^Median and 25th and 75th percentiles; ^¶^The minimum wage at data collection was R$ 1,320.00, Brazil, 2023

**Table 2 -:** Sociodemographic characteristics and health status of elderly stroke survivors* (n = 58). Porto Alegre, RS, Brazil, 2024

**Variables**	**IG** ^†^ **(n=29)**	**CG** ^‡^ **(n=29)**	**p**
Age (years)^‡^	72.9 ± 7.0	71.8 ± 7.3	0.572
Gender - Female^§^	18 (62.1)	13 (44.8)	0.292
Marital status^§^ - With a partner	14 (48.3)	17 (58.6)	0.599
Professional Status^§^ - Without occupation	28 (96.6)	27 (93.1)	1.000
Education^§^			0.180
Elementary school	17 (58.6)	17 (58.6)	
High School	9 (31.0)	12 (41.4)	
Higher education/Post-graduation studies	3 (10.3)	0 (0.0)	
Stroke* – Ischemic^||^	26 (89.7)	26 (89.7)	1.000
Morbidities^||^	28 (96.6)	28 (96.6)	1.000
Cardiovascular diseases	26 (89.7)	25 (86.2)	1.000
Diabetes Mellitus	7 (24.1)	10 (34.5)	0.564
Place of Admission^||^			0.218
SCU^¶^-Stroke*	23 (79.3)	19 (65.5)	
Emergency	5 (17.2)	10 (34.4)	
Admission Unit	1 (3.4)	0 (0.0)	
Total Functional Capacity of FIM**			
Baseline	62.7 ± 31.2	60.0 ± 28.9	0.829^††^
Final	101.8 ± 19.8	90.4 ± 26.7	0.144^††^
Difference	39.1 (28.2 a 50.1)	30.4 (20.9 a 39.9)	0.472^††^
Did the elderly person have any hospital readmissions after discharge^||^	3 (12.0)	5 (20.0)	0.702
Did the elderly person require emergency care^||^	6 (24.0)	6 (24.0)	1.000

*Stroke = Cerebrovascular Accident; ^†^IG = Intervention group; ^‡^CG = Control group; ^§^Mean ± standard deviation; ^||^Absolute number and (%); ^¶^SCU = Special care unit; **FIM = Functional Independence Measure; ^††^Effect of intervention between groups using the Generalized Estimating Equations (GEE) model complemented by the Least Significant Difference (LSD) test, with p-value adjusted for kinship

The effects of the intervention on the CICDEP-STROKE scale scores are presented in Table 3. A statistically significant increase in the total scale score was observed in both groups (p<0.001). However, in the intergroup comparison, there was no statistically significant difference (p=0.604).

In the comparison of the items on the CICDEP-STROKE scale between the groups, significantly higher scores were observed in the question “Helps in administering medications as prescribed by the doctor” in the IG (p=0.006), at the end of the intervention.

In the comparison of the items on the intragroup scale, there was a statistically significant increase in both groups, in the questions: Places food and utensils on the side on which the elderly person is most dependent to stimulate the affected limb; Provides support and/or materials necessary to facilitate eating; Provides support and/or materials necessary to facilitate personal hygiene; Provides privacy when using the toilet, changing diapers or bathing; Provides support and/or materials necessary to facilitate dressing; Explains to the elderly person the correct way to move from one place to another; Provides support and/or materials needed for the elderly person to move from one place to another; Provides support and/or materials needed to position the elderly person.

Only in the IG was there a statistically significant increase in the following items: Controls food intake; Helps administer medications as prescribed by the doctor; Helps the elderly person to move from one place to another; Assesses the need to rotate the elderly person’s body position. On the other hand, there was a statistically significant reduction in the item: Maintains a well-groomed appearance in this group.

There was a statistically significant increase only in the CG in the items: Introduces water if the tube becomes obstructed during the administration of food and medications; Introduces water to wash the tube after the administration of food and medications; Provides support and/or materials needed to facilitate urinary and bowel elimination; Helps with personal hygiene after using the toilet or changing diapers; Assesses the elderly person’s ability to move from one place to another.

A similar improvement was observed in the training of caregivers in both groups. No statistically significant changes were observed in the training scores for the other questions.

**Table 3 -:** Effects of the intervention on the CICDEP-STROKE* scores of family caregivers (n = 58). Porto Alegre, RS, Brazil, 2024

**Items**	**IG** ^†^ **Mean ± SD** ^||^	**CG** ^‡^ **Mean ± SD** ^||^	**p**_adjusted_^§^
Total			
Baseline	2.34 ± 0.44	2.34 ± 0.41	0.812
Final	2.66 ± 0.31	2.72 ± 0.19	0.269
Difference	0.31 (0.15 a 0.48)	0.38 (0.19 a 0.57)	0.604
p	<0.001	<0.001	
Prepares meals according to the prescribed or guided diet.			
Baseline	2.45 ± 0.87	2.69 ± 0.60	0.065
Final	2.68 ± 0.65	2.82 ± 0.39	0.225
Difference	0.23 (-0.11 a 0.58)	0.13 (-0.10 a 0.36)	0.405
p	0.187	0.268	
Prepares the meal in an appropriate manner.			
Baseline	2.48 ± 0.79	2.62 ± 0.56	0.365
Final	2.74 ± 0.54	2.86 ± 0.47	0.581
Difference	0.26 (-0.12 a 0.63)	0.24 (-0.06 a 0.54)	0.944
p	0.181	0.112	
Places food and utensils on the side where the elderly person is most dependent to stimulate the affected limb.			
Baseline	1.61 ± 1.32	1.41 ± 1.09	0.913
Final	2.24 ± 1.09	2.14 ± 1.24	0.781
Difference	0.63 (0.02 a 1.24)	0.73 (0.23 a 1.23)	0.766
p	0.042	0.004	
Provides support and/or materials needed to facilitate eating.			
Baseline	2.56 ± 0.64	2.57 ± 0.63	0.906
Final	2.96 ± 0.20	2.96 ± 0.20	0.979
Difference	0.40 (0.17 a 0.64)	0.39 (0.15 a 0.63)	0.913
p	<0.001	0.002	
Controls food intake.			
Baseline	2.41 ± 1.02	2.59 ± 0.84	0.327
Final	2.87 ± 0.34	2.90 ± 0.31	0.366
Difference	0.46 (0.09 a 0.83)	0.31 (-0.03 a 0.64)	0.547
p	0.016	0.074	
Monitors swallowing.			
Baseline	2.50 ± 1.00	2.63 ± 0.74	0.444
Final	2.79 ± 0.42	2.89 ± 0.32	0.176
Difference	0.29 (-0.09 a 0.67)	0.27 (-0.06 a 0.59)	0.906
p	0.132	0.110	
Helps administer medications according to medical prescription.			
Baseline	2.66 ± 0.72	2.79 ± 0.49	0.537
Final	3.00 ± 0.00	2.78 ± 0.42	0.006
Difference	0.34 (0.09 a 0.60)	-0.01 (-0.28 a 0.26)	0.074
p	0.009	0.940	
Introduces water if the tube becomes obstructed during the administration of diet and medications.			
Baseline	0.60 ± 0.89	0.20 ± 0.45	0.252
Final	2.00 ± 1.73	3.00 ± 0.00	0.364
Difference	1.40 (-0.23 a 3.03)	2.80 (2.45 a 3.15)	0.200
p	0.091	<0.001	
Introduces water to wash the tube after administering diet and medication.			
Baseline	0.60 ± 0.89	0.20 ± 0.45	0.252
Final	2.00 ± 1.73	3.00 ± 0.00	0.364
Difference	1.40 (-0.23 a 3.03)	2.80 (2.45 a 3.15)	0.200
p	0.091	<0.001	
Hydrates the skin.			
Baseline	2.39 ± 0.96	2.39 ± 0.92	0.783
Final	2.41 ± 0.87	2.65 ± 0.70	0.288
Difference	0.02 (-0.56 a 0.60)	0.25 (-0.11 a 0.62)	0.507
p	0.949	0.176	
Prepares hygiene material.			
Baseline	2.72 ± 0.53	2.61 ± 0.74	0.646
Final	2.61 ± 0.85	2.86 ± 0.35	0.202
Difference	-0.11 (-0.52 a 0.29)	0.26 (-0.07 a 0.58)	0.164
p	0.586	0.118	
Provides support and/or materials needed to facilitate personal hygiene.			
Baseline	2.72 ± 0.53	2.45 ± 0.91	0.110
Final	3.00 ± 0.00	2.96 ± 0.21	0.177
Difference	0.28 (0.09 a 0.46)	0.51 (0.16 a 0.85)	0.222
p	0.004	0.004	
Helps with bathing.			
Baseline	2.52 ± 0.74	2.41 ± 0.80	0.463
Final	2.50 ± 0.91	2.68 ± 0.48	0.626
Difference	-0.02 (-0.59 a 0.56)	0.28 (-0.09 a 0.65)	0.417
p	0.953	0.141	
Helps with oral hygiene.			
Baseline	2.55 ± 0.78	2.69 ± 0.55	0.676
Final	1.86 ± 1.35	2.86 ± 0.36	0.065
Difference	-0.69 (-1.67 a 0.28)	0.16 (-0.14 a 0.47)	0.168
p	0.162	0.296	
Maintains a well-groomed appearance.			
Baseline	2.93 ± 0.26	2.69 ± 0.71	0.048
Final	2.67 ± 0.58	2.75 ± 0.55	0.743
Difference	-0.26 (-0.51 a -0.01)	0.06 (-0.31 a 0.43)	0.164
p	0.038	0.752	
Provides privacy when using the toilet. changing diapers or bathing.			
Baseline	2.46 ± 0.84	2.54 ± 0.79	0.532
Final	2.82 ± 0.73	2.88 ± 0.33	0.546
Difference	0.36 (0.06 a 0.66)	0.35 (0.01 a 0.69)	0.966
p	0.018	0.046	
Provides support and/or materials needed to facilitate urinary and bowel elimination.			
Baseline	2.48 ± 0.80	2.30 ± 0.72	0.473
Final	2.79 ± 0.71	2.95 ± 0.22	0.337
Difference	0.31 (-0.10 a 0.71)	0.65 (0.35 a 0.95)	0.199
p	0.138	<0.001	
Helps with personal hygiene after using the toilet or changing diapers.			
Baseline	2.22 ± 0.97	2.20 ± 0.91	0.751
Final	2.25 ± 1.17	2.85 ± 0.38	0.395
Difference	0.03 (-0.74 a 0.79)	0.65 (0.25 a 1.05)	0.297
p	0.943	0.002	
Provides support and/or materials needed to make dressing easier.			
Baseline	2.57 ± 0.69	2.69 ± 0.54	0.561
Final	3.00 ± 0.00	2.91 ± 0.29	0.178
Difference	0.43 (0.18 a 0.68)	0.22 (0.01 a 0.43)	0.254
p	<0.001	0.037	
Helps the person get dressed.			
Baseline	2.46 ± 0.79	2.70 ± 0.54	0.153
Final	2.72 ± 0.46	2.71 ± 0.46	0.811
Difference	0.26 (-0.06 a 0.58)	0.01 (-0.28 a 0.30)	0.289
p	0.115	0.942	
Assesses the elderly person’s ability to transfer from one place to another.			
Baseline	2.28 ± 0.99	2.36 ± 0.95	0.724
Final	2.67 ± 0.70	2.82 ± 0.50	0.311
Difference	0.39 (-0.07 a 0.85)	0.46 (0.06 a 0.86)	0.774
p	0.095	0.025	
Explains to the elderly person the correct way to transfer from one place to another.			
Baseline	2.24 ± 0.91	2.00 ± 1.07	0.639
Final	2.71 ± 0.69	2.65 ± 0.57	0.849
Difference	0.47 (0.09 a 0.84)	0.65 (0.21 a 1.10)	0.594
p	0.015	0.004	
Provides support and/or materials needed for the elderly person to transfer from one place to another.			
Baseline	2.17 ± 0.97	2.03 ± 0.94	0.729
Final	2.86 ± 0.64	2.95 ± 0.22	0.403
Difference	0.69 (0.37 a 1.02)	0.92 (0.55 a 1.28)	0.344
p	<0.001	<0.001	
Helps the elderly person to transfer from one place to another.			
Baseline	2.21 ± 0.98	2.39 ± 0.74	0.268
Final	2.67 ± 0.73	2.76 ± 0.56	0.504
Difference	0.46 (0.10 a 0.82)	0.37 (-0.05 a 0.80)	0.658
p	0.011	0.085	
Uses appropriate posture to transfer the elderly person from one place to another.			
Baseline	1.61 ± 1.07	1.68 ± 0.98	0.481
Final	1.95 ± 1.00	2.00 ± 0.86	0.659
Difference	0.35 (-0.11 a 0.81)	0.32 (-0.11 a 0.75)	0.766
p	0.138	0.142	
Provides support and/or materials needed to position the elderly person.			
Baseline	2.15 ± 0.91	2.36 ± 0.91	0.398
Final	2.94 ± 0.24	2.80 ± 0.52	0.300
Difference	0.80 (0.48 a 1.12)	0.44 (0.02 a 0.86)	0.222
p	<0.001	0.040	
Assesses the need to alternate the elderly person’s body position.			
Baseline	2.29 ± 0.90	2.31 ± 0.85	0.961
Final	2.65 ± 0.61	2.43 ± 0.65	0.275
Difference	0.36 (0.05 a 0.67)	0.12 (-0.32 a 0.55)	0.353
p	0.022	0.593	
Uses appropriate posture to position each part of the elderly person’s body correctly.			
Baseline	1.74 ± 1.13	1.61 ± 1.07	0.646
Final	2.17 ± 0.79	1.95 ± 0.83	0.395
Difference	0.43 (-0.12 a 0.98)	0.34 (-0.11 a 0.80)	0.898
p	0.129	0.138	
Reverses the elderly person’s body position when they are lying down.			
Baseline	2.16 ± 1.07	2.13 ± 0.95	0.901
Final	2.57 ± 0.79	2.11 ± 0.93	0.251
Difference	0.41 (-0.31 a 1.13)	-0.01 (-0.75 a 0.72)	0.417
p	0.262	0.970	

*CICDEP-STROKE = Scale of Capabilities of Informal Caregivers for Dependent Elderly People due to Stroke; ^†^GI = Intervention group; ^‡^CG = Control group; ^§^Effect of the intervention between groups by the Generalized Estimating Equations (GEE) model complemented by the Least Significant Difference (LSD) test, with p-value adjusted for kinship; ^¶^Mean ± standard deviation

The effect of the intervention on family caregiver burden is presented in Table 4. There was no significant difference between the groups. Regarding total burden, a statistically significant reduction was observed in both groups (p<0.001). In the intragroup comparisons of the domains, there was a statistically significant reduction in the domains of general tension, isolation and environment in both groups. In the disappointment domain, there was a significant reduction only in IG (p=0.011).

**Table 4 -:** Effects of the intervention on the CBS* scores of family caregivers (n = 58). Porto Alegre, RS, Brazil, 2024

**Domains**	**IG** ^†^ **Mean ± SD** ^||^	**CG** ^‡^ **Mean ± SD** ^||^	**p**_adjusted_^§^
CBS* total			
Basal	1.87 ± 0.46	2.01 ± 0.51	0.570
Final	1.53 ± 0.43	1.60 ± 0.51	0.912
Difference	-0.34 (-0.52 a -0.17)	-0.41 (-0.64 a -0.18)	0.679
p	<0.001	<0.001	
General tension			
Basal	2.02 ± 0.65	2.12 ± 0.76	0.916
Final	1.69 ± 0.68	1.64 ± 0.69	0.533
Difference	-0.33 (-0.64 a -0.03)	-0.48 (-0.82 a -0.14)	0.542
p	0.033	0.006	
Isolation			
Basal	1.67 ± 0.62	2.06 ± 1.01	0.099
Final	1.24 ± 0.43	1.46 ± 0.74	0.296
Difference	-0.43 (-0.71 a -0.15)	-0.60 (-0.94 a -0.26)	0.443
p	0.003	<0.001	
Disappointment			
Basal	1.90 ± 0.75	1.83 ± 0.62	0.417
Final	1.54 ± 0.47	1.57 ± 0.61	0.841
Difference	-0.36 (-0.64 a -0.08)	-0.26 (-0.54 a 0.02)	0.555
p	0.011	0.069	
Emotional involvement			
Basal	1.14 ± 0.24	1.27 ± 0.63	0.462
Final	1.20 ± 0.38	1.16 ± 0.36	0.467
Difference	0.06 (-0.04 a 0.16)	-0.11 (-0.37 a 0.15)	0.227
p	0.254	0.392	
Environment			
Basal	2.26 ± 0.63	2.50 ± 0.79	0.409
Final	1.70 ± 0.57	1.94 ± 0.72	0.350
Difference	-0.56 (-0.84 a -0.28)	-0.56 (-0.90 a -0.21)	0.974
p	<0.001	0.001	

*CBS = Caregiver Burden Scale; ^†^IG = Intervention group; ^‡^CG = Control group; ^§^ Effect of intervention between groups using the Generalized Estimating Equations (GEE) model complemented by the Least Significant Difference (LSD) test, with p-value adjusted for kinship; ^||^Mean ± standard deviation

Regarding course access data, the average total number of accesses was 10.3 (± 5.4) and the average number of modules accessed was 7.8 (± 2.5). There was no statistically significant interference of the total number of accesses (p=0.164) and the number of modules accessed (p=0.182) in the change in CICDEP-STROKE scores in the IG. The most accessed module was Module 1 - What is a Stroke (36 accesses), followed by Module 4 - Medication care (32 accesses). The least accessed was Module 11 - Tracheostomy care (14 accesses).

## Discussion

This RPE analysed the effects of a virtual educational intervention aimed at family caregivers of elderly stroke survivors. A similarity was observed between the two groups, with regard to improved training and reduced caregiver burden. The main result of this study states that the intervention significantly benefited the caregivers who received it in improving their ability to administer medications.

Regarding the characterization of the participants, the majority of caregivers were women, with an average age of around 50 years and a higher proportion of children in the CG when compared to the IG, which is noteworthy because, in Brazil, the role of caring for dependent elderly people is historically directed to daughters and wives/partners^(24)^. This data may have interfered with the results obtained, to the extent that children assume responsibility for care because they feel obliged to do so, but the feeling of duty can transform into motivation and inspiration to care for elderly parents, despite the difficulties in adapting to this new role^(25)^.

Furthermore, regarding the characteristics of the participants, it was observed that there were more caregivers in the CG who had received formal training to provide care prior to the study. Furthermore, in this group, 75.9% of the participants had already cared for another dependent person, and the same situation was observed in 69% of the caregivers in the IG. These data indicate that, possibly, the caregivers studied had already developed some self-confidence regarding their competence, knowledge and skills to perform daily tasks and deal with their emotions^(26)^, suffering less interference from the intervention.

Regarding the characteristics of the elderly, the averages of the FIM scale indicated that the stroke survivors were less dependent in the final evaluation for both groups, although there was no statistical significance, which can be justified considering that most of them had an ischemic event, which tends to generate less dependence over time when compared to haemorrhagic events^(27)^.

When analysing each item of the CICDEP-STROKE scale, an improvement was observed in four items for the IG and in five items for the CG, indicating a similar increase in the caregivers’ training, which can be attributed to their own care experience. In the item on medication administration, the IG caregivers had better results at the end of the intervention, even though the change between the groups was not significant. The module on medication care was the most accessed by caregivers, second only to the introductory module on what stroke is. A study with family caregivers of dependent elderly people identified that medication care represented one of the main activities that was difficult to perform, due to the lack of information and knowledge for its performance^(28)^.

The Portuguese study that developed the original instrument *Escala de Capacities do Prestador Informal de Cuidados de Idosos Dependentes por AVC* (ECPICID-AVC), later validated for use in Brazil as CICDEP-STROKE^(19)^, applied a training program called Intervention in Informal Caregivers who take Care of Older People after a Stroke (InCARE), in which 85 family caregivers of elderly people who survived stroke received guidance aimed at increasing knowledge and practical skills, through home visits (HVs) and telephone calls, for three months after discharge. The control group (n = 89) received usual health care. The results indicated that the experimental group obtained a higher mean CICDEP-STROKE score over time and decreased burden^(29)^. To date, no other studies have been found that used CICDEP-STROKE to assess the training of caregivers after an intervention.

However, several intervention studies dedicated to improving the training of family caregivers of dependent individuals due to stroke have been identified in the literature. A Chinese randomized clinical trial (RCT) evaluated the effects of a psychoeducational intervention (Family-focused dyadic psychoeducational intervention – FDPEI) on caregiver burden and competence. The pre-discharge phase included three in-person sessions and aimed to prepare for the transition from hospital to home; the post-discharge phase included four weekly counselling phone calls, encouraging caregivers to identify difficulties and motivate them to deal with them. A significant reduction in burden and improvement in caregiver competence to care were observed^(30)^.

A Thai study also offered an intervention, carried out by nurses, which consisted of sessions aimed at increasing caregivers’ behavioural skills based on information and motivation. Lasting eight weeks, the intervention included a pre-discharge phase of guidance on stroke and 2 post-discharge HVs. The intervention improved the caregiving skills of family caregivers^(31)^.

Unlike the e-share intervention, the FDPEI program and the Thai study conducted multiple face-to-face sessions before discharge. It is known that for home care to be carried out in a way that meets the needs of elderly stroke patients and reduces the impact on caregivers, it must begin to be prepared and planned before hospital discharge^(8)^. Conducting only one face-to-face session before discharge may not have been sufficient to obtain better results in this study.

A study conducted in Hong Kong applied an intervention carried out by a multidisciplinary team through a 26-week educational program. The intervention group demonstrated significant improvements in terms of care competence and a lower level of burden three months after the intervention^(32)^. A study conducted in Peru evaluated the effect of eight educational videos with themes on positioning, mobilization and transfers, prepared by a multidisciplinary team, to improve the practical skills and knowledge of ten informal caregivers of stroke patients. The scores for practical skills and knowledge increased significantly^(33)^.

The studies conducted in Hong Kong and Peru offered a combination of care strategies through a multidisciplinary team; these different approaches may explain the lack of differences in the scores of caregivers’ training and burden in e-share. However, regarding the study conducted in Peru, the limitations of the researchers not having evaluated the caregivers after the care experience and the small sample size are noteworthy.

Regarding the burden faced by family caregivers of elderly stroke survivors, it is well documented in the literature; especially with regard to the transition to home care^(34)^, it is expected that better trained caregivers will experience less burden. A Nigerian study evaluated the moderating role of care preparation on family caregiver burden. The results showed that preparation moderated the relationship between burden and caregivers’ physical and mental health^(35)^.

Ensuring that family caregivers are adequately prepared and have support can reduce the negative impacts of caregiving^(35)^. Among the e-share caregivers, a reduction in total burden was observed in both groups, and although there was no effect of the intervention between groups, there was a significant reduction in the “Disappointment” domain only in the IG, which can be explained by the fact that the intervention offered availability of contact, reinforced the importance of the involvement of all family members in care and that the primary caregiver needed moments of rest, information contained in the MOOC in Module 2.

Although no significant interference was found from access to the MOOC or change in CICDEP-STROKE scores, there was coherence between the most accessed modules and the identified care needs.

A pilot RCT study conducted in the United States evaluated the feasibility and acceptability of an intervention for support and problem-solving via the internet and telephone for caregivers of stroke survivors. The two-arm intervention, delivered by nurses, was based on the RESCUE website, which includes the following sections: (1) information sheets, (2) list of additional resources; (3) self-management; (4) glossary of stroke-related terms; (5) testimonials; (6) training module; and (7) problem-solving diary. The intervention was delivered by telephone in four or eight weekly sessions lasting 30-60 minutes each, tailored to the problems of each caregiver, for eight weeks after hospital discharge. The results indicated positive responses to the intervention, and qualitative analysis revealed that the intervention was valued and acceptable by caregivers^(36)^.

It is noteworthy that the aforementioned study, with significant effects, was developed in a country with more favourable social and economic conditions, differing from the characteristics found in Brazil. Even so, it is worth noting that the use of these modalities contributes to the universalization of digital access and health information.

In the last 15 years, educational interventions for caregivers have been carried out in real environments, and with the 2019 coronavirus pandemic, research in virtual environments has been intensified. A systematic review with meta-analysis sought to elucidate the evidence related to the use of e-Health (digital and/or virtual tools and solutions aimed at health) in improving the QoL of informal caregivers of dependent stroke patients. The analysis concluded that there was a trend towards improvement in the mental health of caregivers; improved ability to solve problems related to care; and prevention of problems resulting from overload^(10)^.

This RPE has limitations. Most of the elderly stroke survivors were recruited from the UCE-Stroke (23 IG vs. 19 CG), a place where caregivers also had the support of health professionals with highly specialized knowledge. This fact, associated with the sample characteristic in which participants in both groups had previous experience as caregivers, may have contributed to our finding non-significant results. In a future study, a more inclusive sample of elderly individuals from non-specialized institutions is needed.

It is inferred that the evaluation of outcomes over a longer follow-up period may also interfere with the results, since the care experience itself may have favoured the similarity of results between the groups. In Brazil, nurses conducted the Nursing Home Care Intervention Post Stroke (SHARE) RCT, which consisted of preparing caregivers to perform ADLs for elderly individuals who suffered a stroke after discharge. The results demonstrated similar QoL and an increase in total burden in the CG and IG. However, regarding QoL, caregivers in the IG presented higher scores for social relations, and regarding burden, the IG exhibited less isolation; the outcome assessment occurred 60 days and one year after hospital discharge^(37-38)^.

Furthermore, the lack of research for the purpose of comparing changes in CICDEP-STROKE scores is highlighted as a limitation. We hope that this study encourages researchers to adopt its use, as well as the expansion of the use of virtual technologies, in Brazilian investigations.

In addition to research, this study has practical application; the data will be disseminated to the health service and the MOOC will be available to CG caregivers and to the population, on an open platform. In this way, it is expected to support family caregivers who do not have access to health services with specialized teams.

This is the first study in Brazil that proposes the use of MOOC as an educational tool for family caregivers of elderly stroke survivors, representing an important advance for Nursing in the construction of digital educational technologies.

## Conclusion

The multicomponent intervention, e-share, provided, in addition to the MOOC, a telephone line and four scheduled contacts, but, even so, no effect of the intervention was observed in improving the training and reducing the burden of caregivers. However, it significantly favoured IG caregivers in terms of improving medication administration and reducing the burden in the disappointment domain.

This study reveals the complexity of care transition actions after a stroke. The use of simple virtual technologies explores alternatives for this transition that provide support to caregivers in acquiring skills and knowledge. It is necessary that caregivers be evaluated before the elderly person is discharged from hospital, ensuring an adequate transition and reducing the negative effects of care, highlighting the central role of the nurse in these actions.
