# Supplementary material for: Effectiveness of e-share intervention for caregivers of elderly people after stroke: a pragmatic randomized trial
Source: Rev Lat Am Enfermagem. 2025 Feb 17;33:e4467. doi: 10.1590/1518-8345.7414.4467 (PMC11835008; doi:10.1590/1518-8345.7414.4467)
Supplement: Translation

# Translation: Efetividade da intervenção *e-share* para cuidadores de idosos após acidente vascular cerebral: ensaio pragmático randomizado *

**DOI:** 10.1590/1518-8345.7414.4468

**Authors:** Francine Melo da Costa, Débora Francisco do Canto, Lisiane Manganelli Girardi Paskulin

**Affiliations:** 1 Universidade Federal do Rio Grande do Sul, Escola de Enfermagem, Porto Alegre, RS, Brasil; 2 Hospital de Clínicas de Porto Alegre, Serviço de Enfermagem Ambulatorial, Porto Alegre, RS, Brasil; 3 Hospital de Clínicas de Porto Alegre, Serviço de Enfermagem Clínica, Porto Alegre, RS, Brasil; 4 Hospital de Clínicas de Porto Alegre, Diretoria de Enfermagem, Porto Alegre, RS, Brasil; 5 Bolsista do Conselho Nacional de Desenvolvimento Científico e Tecnológico (CNPq), Brasil

**Keywords:** Cuidadores, Educação, Acidente Vascular Cerebral, Transição do Hospital para o Domicílio, Idoso, Tecnologia Educacional

## Abstract

**Objetivo:**

analisar a efetividade de uma intervenção educativa virtual para cuidadores familiares na sobrecarga e na capacidade de cuidarem de idosos após acidente vascular cerebral.

**Método:**

ensaio pragmático randomizado com 58 cuidadores de idosos sobreviventes de acidente vascular cerebral. O grupo de intervenção recebeu acesso a um curso *online* para educação dos cuidadores. A capacitação do cuidador foi avaliada através da Escala de Capacidades do Cuidador Informal de Idosos Dependentes por Acidente Vascular Cerebral e a sobrecarga através da *Caregiver Burden Scale* antes da alta hospital e, também, três meses após, ambas foram adaptadas e validadas para uso no Brasil. Ainda, foi adotado o modelo de Equações de Estimativas Generalizadas complementado pelo *Least Significant Difference*.

**Resultados:**

a amostra foi homogênea e os grupos diferiram estatisticamente apenas em relação ao parentesco (p=0,034), observada maior proporção de filhos no grupo controle. Houve efeito da intervenção na melhora da administração dos medicamentos (p=0,006) e na redução da decepção (p=0,011) no grupo intervenção.

**Conclusão:**

a intervenção favoreceu os cuidadores que a receberam, no que se refere à melhora da administração de medicamentos e à redução da decepção. No Brasil, este é o primeiro estudo que propõe o uso de tecnologias educacionais digitais para esse grupo, representando importante avanço da Enfermagem. Registro clinicaltrial.gov: NCT05553340.

## Destaques

**(1)** Primeiro estudo, no Brasil, que propõe um MOOC para cuidadores de idosos após AVC. **(2)** Avanço da Enfermagem na construção de tecnologias educacionais digitais. **(3)** A *e-share* favoreceu os cuidadores na melhora da administração de medicamentos. **(4)** A *e-share* favoreceu os cuidadores na redução da sobrecarga no domínio decepção. **(5)** Ferramentas digitais contribuem para o aproveitamento do trabalho dos enfermeiros.

## Introdução

O Acidente Vascular Cerebral (AVC) é uma condição neurológica que acomete principalmente pessoas idosas, sendo a segunda maior causa de morte no Brasil e no mundo^(1)^. No Brasil, entre janeiro e outubro de 2023, cerca de 150 mil pessoas foram internadas por AVC, das quais, aproximadamente, 117 mil (79%) tinham 60 anos ou mais e em torno de 18 mil (28%) foram a óbito^(2)^. Além da alta taxa de mortalidade, o AVC é considerado uma das principais causas de incapacidade na população idosa. Os idosos que sofrem um AVC têm mais limitações para o autocuidado após a alta hospitalar, necessitando de auxílio^(1)^.

O cuidado informal envolve inúmeras tarefas, como apoio prático, emocional, financeiro e social^(3)^. No Brasil, a disponibilidade de apoio de redes formais é restrita, recaindo sobre a família a responsabilidade pela maior parte dos cuidados^(4)^. Além disso, o suporte ao cuidador informal não é explicitado nas políticas públicas^(5)^, deixando as famílias com a obrigação de cuidar e muitas vezes sem informações, qualificação e recursos necessários^(6)^.

De modo específico, sobre os cuidadores informais de idosos dependentes por AVC, um estudo identificou que as principais atividades de cuidado executadas eram: fornecer materiais e/ou apoio para alimentar-se, vestir-se e cuidar das medicações. Já as atividades nas quais os cuidadores apresentaram maior dificuldade foram transferência e posicionamento^(7)^.

Os estudos supracitados expõem a existência de lacunas na qualidade do atendimento oferecido para as famílias. Em contrapartida, recomendações internacionais para a transição do cuidado do hospital para o domicílio apontam que a educação dos cuidadores familiares deve incluir o treinamento de técnicas de cuidados pessoais, como alimentação, transferência e posicionamento^(8)^.

Ainda, estudos internacionais têm demonstrado que intervenções educativas direcionadas a cuidadores familiares de idosos após um AVC auxiliam na redução da sobrecarga^(9)^, aprimoram a capacidade para assistir o idoso no domicílio^(9)^, melhoram a Qualidade de Vida (QV) do cuidador^(8)^ e impactam positivamente a saúde física e mental dos sobreviventes^(8)^.

Entretanto, ainda não há evidências, no contexto nacional e em condições mais próximas àquelas encontradas na prática de cuidado, de que uma intervenção educativa baseada no desenvolvimento de habilidades e de conhecimentos possa melhorar a capacidade dos cuidadores para realizar a atenção ao idoso. Ademais, intervenções virtuais voltadas aos cuidadores são alternativas recentes que merecem ser mais bem testadas^(10)^.

Logo, a presente investigação foi desenvolvida considerando a hipótese de que a intervenção educativa virtual, realizada por enfermeiros para cuidadores familiares de idosos após AVC, melhora a capacidade de cuidar, quando comparada ao acompanhamento usual. O conceito de “capacidade” adotado neste estudo provém da Teoria Social Cognitiva, na qual o autor traduz conhecimentos, habilidades e competências como resultado do termo “capacidade”^(11-12)^.

Ademais, este estudo explora alternativas de transição do cuidado, a otimização dos recursos, a melhoria na experiência do cuidado e o melhor aproveitamento do tempo de trabalho dos enfermeiros, bem como evidencia e fortalece o seu papel educador. Objetivou-se analisar a efetividade de uma intervenção educativa virtual para cuidadores familiares, na sobrecarga e na capacidade de cuidarem de idosos após acidente vascular cerebral, quando comparada com orientações usuais de cuidado, no período de três meses após alta hospitalar.

## Método

### Delineamento

Estudo Pragmático Randomizado (EPR), denominado *Educational Intervention With Digital Technology For Family Caregivers* (*e-share*), cadastrado no *clinicaltrial.gov* sob o número NCT05553340. A pesquisa seguiu as recomendações da extensão do *Consolidated Standards of Reporting Trials* (CONSORT)^(13)^.

### Local e período

O estudo foi desenvolvido no Serviço de Emergência, nas cinco unidades de internação clínica e na Unidade de Cuidados Especiais de AVC (UCE-AVC), de um hospital universitário de grande porte no Sul do Brasil.

A UCE-AVC atende pacientes com diagnóstico de AVC, possui 10 leitos e oferece acompanhamento por equipe multidisciplinar composta por médicos, enfermeiros, farmacêuticos, nutricionistas, fisioterapeutas, fonoaudiólogos, assistentes sociais e psicólogos. Na alta, esses profissionais oferecem aos pacientes, e a seus familiares, informações acerca da doença, além de materiais educativos referentes aos cuidados que serão realizados no domicílio. Conforme a lotação desses leitos ou a gravidade dos pacientes, estes podem ser alocados em outras unidades de internação ou ter alta diretamente da emergência^(14)^.

Vale evidenciar que o recrutamento ocorreu no período de janeiro a junho de 2023, e a coleta de dados, de janeiro a novembro do mesmo ano.

### População e amostra

Os participantes do estudo foram os cuidadores familiares de idosos com 60 anos ou mais. O cuidador familiar é entendido como a pessoa responsável pelos cuidados ao idoso no domicílio, não remunerado e que pode ou não ser um membro da família^(15)^.

Foram incluídos os cuidadores familiares com idade acima de 18 anos e que exerciam o papel de principal provedor dos cuidados, não remunerado, aos pacientes com 60 anos ou mais (de ambos os sexos), com diagnóstico médico de AVC na internação atual. Foram excluídos os cuidadores familiares que: (a) não tinham acesso à internet; (b) não estivessem aptos a acessar a intervenção virtual, verificado através do *checklist* de aptidão para acesso e navegação ao curso, elaborado para este estudo; (c) não tinham linha telefônica para contato; (d) estavam acompanhando idosos que fossem transferidos para instituições de longa permanência após a alta; (e) estavam acompanhando idosos que vieram a óbito, na fase de captação dos participantes.

Além disso, foi calculado o tamanho de amostra para detectar diferenças na média da escala utilizada para avaliação da capacitação dos cuidadores, entre os grupos intervenção e controle, por meio da ferramenta *Power and Sample Size for Health Researchers* (*PSS Health*)^(16)^. Considerou-se poder de 80%, nível de significância de 5% e tamanho de efeito de Cohen de 0,8. Desse modo, quanto maior o valor do d de Cohen, mais significativa é a discrepância entre as médias, valores de d de 0,8 representam efeitos grandes^(17)^. Este foi adotado, uma vez que, no momento da execução desta pesquisa, não havia estudos prévios que aplicassem a escala adotada, buscando identificar diferenças nas médias.

A estimativa do tamanho total da amostra atingiu 52 participantes, 26 em cada grupo. Somando 10% para possíveis recusas, o tamanho amostral foi de 58 participantes, sendo 29 em cada grupo. Os participantes elegíveis foram os cuidadores familiares de pacientes com 60 anos ou mais, com diagnóstico médico de AVC, internados nas unidades da instituição e monitorados por prontuários eletrônicos disponíveis no*software*institucional, no período da coleta de dados.

### Variáveis e instrumentos

O desfecho primário foi a capacidade dos cuidadores informais para cuidar de idosos após AVC e o desfecho secundário foi a sobrecarga do cuidador; os participantes foram avaliados no momento da avaliação basal, ou seja, na internação hospitalar, e na avaliação final, a saber, 90 dias após a alta.

Foram coletadas informações sociodemográficas e clínicas dos idosos e de seus cuidadores familiares. Para os sobreviventes de AVC, também foi avaliada a Medida da Independência Funcional (MIF). A MIF avalia quantitativamente a carga de cuidados que um paciente dependente demanda para outra pessoa na realização de tarefas motoras e cognitivas. Ademais, é composta por 10 categorias divididas em 6 dimensões: autocuidado, controle de esfíncteres, transferências, locomoção, comunicação e cognição social. As pontuações gerais da MIF podem variar de 18 a 126. Logo, quanto menor o escore, maior a dependência do idoso para a realização das tarefas. A escala passou por processo de validação no contexto brasileiro, em 2004, apresentando boa correlação no teste/reteste e na reprodutibilidade interobservadores (Pearson: 0,91-0,98; coeficiente de correlação intraclasse – ICC: 0,91-0,98 e Pearson: 0,87 – 0,98; ICC: 0,87 – 0,98, respectivamente)^(18)^.

O desfecho primário foi verificado através da Escala de Capacidades do Cuidador Informal de Idosos Dependentes por AVC (ECCIID-AVC), que avalia as diferentes capacidades que os cuidadores familiares têm ou que precisam melhorar na prestação de cuidados a idosos dependentes após um AVC. Adaptada e validada para uso no Brasil, a ECCIID-AVC apresenta confiabilidade teste-reteste satisfatória (ICC = 0,94; Intervalo de confiança de 95% = 0,91-0,96) e uma excelente confiabilidade de consistência interna (alfa de Cronbach = 0,914)^(19)^.

Ainda, a escala é composta de 29 itens que abordam questões relacionadas às seguintes atividades: comer/beber (via oral ou sonda nasogástrica/gastrostomia), administração de medicamento, cuidados de higiene (banho e eliminações), cuidados com a pele, vestir/despir e transferência/posicionamento. Estes itens são avaliados em uma série de 0 (não demonstra - não desempenha a atividade) a 3 (demonstra totalmente - desempenha a atividade de forma correta e autônoma). Além dessas, a opção de resposta “NA – não se aplica” pode ser utilizada nos casos em que o cuidador não realiza determinada atividade, pois o idoso não necessita do cuidado^(19)^. A pontuação total da escala varia de 0 a 87 pontos, não havendo um ponto de corte, e quanto maior a pontuação, mais capacitado o cuidador estará^(19)^.

O desfecho secundário foi avaliado através da escala *Caregiver Burden Scale* (CBS)^(20)^, que possui 22 itens divididos em 5 dimensões, a saber: tensão geral, isolamento, decepção, envolvimento emocional e ambiente. Podem ser atribuídas às questões respostas de 1 - de modo algum a 4 - frequentemente. O escore total é obtido pela média aritmética dos valores equivalentes às respostas das 22 questões, já o escore individual é obtido a partir da média dos valores equivalentes às respostas das questões de cada dimensão. Não há ponto de corte; quanto maior o escore, maior a sobrecarga^(20)^. A escala foi adaptada e validada ao contexto brasileiro, os coeficientes de reprodutibilidade intra e interobservador foram 0.87 e 0.92, respectivamente^(20)^.

### Coleta de dados

A coleta de dados iniciou-se com a identificação dos pacientes com 60 anos ou mais, com diagnóstico médico de AVC na internação, por meio de consulta diária ao sistema informatizado do hospital. Os idosos captados receberam a visita dos pesquisadores avaliadores para verificar a existência de cuidador familiar e se ele e o idoso atendiam aos critérios de inclusão do estudo, e procedendo-se à aplicação do *checklist* de aptidão para acesso ao curso. Caso o cuidador presente neste momento não fosse o cuidador principal, era feito contato telefônico para averiguar se os critérios de inclusão eram atendidos, além disso, era agendada a primeira avaliação. Os que aceitaram participar, assinaram o Termo de Consentimento Livre e Esclarecido (TCLE) e, na sequência, foram coletados os dados basais.

Três meses após alta (90 dias), o cuidador foi contatado via telefone para a realização da avaliação final. A ligação era realizada pelos mesmos pesquisadores responsáveis pela avaliação inicial. Já a coleta se deu via conferência *online* por meio do *Google Meet*® ou por chamada de vídeo via telefone. O intervalo de três meses atendeu ao recomendado pelo estudo de reprodutibilidade brasileira da escala ECCIID-AVC^(19)^, e por evidências quanto à eficácia da implementação dos programas de intervenção educativa, que sugerem pelo menos três meses de intervalo entre as avaliações^(8)^.

Ademais, foi realizada a avaliação da concordância interavaliadores das escalas em estudo antes e após o treinamento para a coleta de dados. Também foi adotado o ICC maior que 0,70 e nível de significância de 5% (p<0,05), e as análises foram realizadas no programa *Statistical Package for the Social Sciences* (SPSS), versão 27.0. Após o treinamento, todos os ICC foram maiores do que 0,70 e encontraram significância estatística (p<0,05).

### Randomização e cegamento

Após a aplicação dos instrumentos, os participantes eram randomizados para grupo controle (GC) ou grupo intervenção (GI). Para tal, foi utilizada uma lista gerada pela página eletrônica https://www.randomizer.org/, que segue uma ordem numérica na qual cada número já está aleatoriamente designado para um dos grupos. Os pesquisadores avaliadores faziam contato com um profissional externo ao grupo de pesquisa responsável pela alocação.

Os pesquisadores que realizaram a avaliação inicial, final e a captação dos participantes foram cegos para a alocação. Na intervenção, o cuidador familiar foi comunicado de que receberia a avaliação final e foi orientado a não mencionar que recebeu a intervenção educativa. Da mesma forma, foi orientado ao GC, por meio de contato telefônico por um membro externo ao grupo de pesquisa, que não informassem ao avaliador que não receberam a intervenção. Sendo assim, as pesquisadoras intervencionistas conheceram apenas os participantes do GI.

### Intervenção

A intervenção multicomponente visou instrumentalizar o cuidador familiar para assistir o idoso nas atividades de vida diária (AVDs) após a alta, e foi realizada por duas enfermeiras - pesquisadoras intervencionistas -, por meio de um curso massivo aberto e *online* (MOOC). Salienta-se que detalhes sobre a elaboração, a construção e o conteúdo do MOOC podem ser vistos em artigo previamente publicado^(21)^.

Além da disponibilização do MOOC, compôs a intervenção a realização de contatos telefônicos com os participantes em 7, 30, 60 e 80 dias após a alta hospitalar, para verificar o andamento do curso e sanar possíveis dificuldades. Também foi disponibilizada uma *hotline* para os participantes, de segunda a sexta-feira, das 8:00 às 18:00, atendida pelas enfermeiras, caso houvesse dúvidas para acesso e navegação no curso. A intervenção teve duração de três meses.

Os incluídos no GI, após a alocação, receberam a visita de instrução, pelas pesquisadoras intervencionistas, ainda na internação hospitalar, com o objetivo de fornecer orientações detalhadas sobre a intervenção. Além disso, os cuidadores eram orientados sobre os contatos telefônicos e a disponibilidade da *hotline*. Neste momento, era entregue um panfleto contendo as orientações, que também foi enviado por *WhatsApp*®. 

### Controle

Para o grupo de controle, não foi oferecida a intervenção. O paciente e seu cuidador familiar receberam orientações de cuidado usuais e o acompanhamento convencional na rede de serviços a que eles têm acesso (pública ou privada), assim como os participantes do GI.

### Análise de dados

Foi adotada a técnica por intenção de tratar^(17)^. Para comparar médias, o teste t de Student foi aplicado. Em caso de assimetria, o teste de Mann-Whitney foi utilizado. Na comparação de proporções, os testes qui-quadrado de Pearson ou exato de Fisher foram aplicados.

Para comparar as escalas intergrupos e intragrupo simultaneamente, o modelo de Equações de Estimativas Generalizadas (GEE) complementado pelo teste *Least Significant Difference* (LSD) foi utilizado. Já o modelo linear foi aplicado para as variáveis numéricas com distribuição normal e o modelo logarítmico para aquelas com distribuição assimétrica. No mesmo modelo foi ajustada a diferença de parentesco entre os dois grupos e o número total de acessos e o de módulos acessados para o GI. O nível de significância adotado foi de 5% (p<0,05) e as análises foram realizadas no programa SPSS, versão 27.0.

### Aspectos éticos

Foram observadas as normas da Resolução nº 466/2012, do Conselho Nacional de Saúde^(22)^, e o Ofício Circular nº 2/2021/CONEP/SECNS/MS, sobre pesquisas em ambiente virtual^(23)^. Os participantes assinaram o TCLE. A pesquisa foi aprovada por Comitê de Ética em Pesquisa, Certificado de Apresentação de Apreciação Ética (CAAE): 59589922.0.0000.5327; e o protocolo de pesquisa foi cadastrado no *clinicaltrial.gov* sob identificação NCT05553340.

## Resultados

Durante o período de recrutamento, 280 cuidadores foram avaliados para elegibilidade. Destes, 222 foram excluídos, sendo: 129 por não atenderem ao critério de inclusão “cuidar de idosos com diagnóstico de AVC confirmado na internação atual”; 15 por apresentarem, pelo menos, um critério de exclusão; e 78 por outros motivos, a saber: a) nove por não aceitarem participar do estudo; e b) 69 por não se ter obtido contato com o cuidador após três tentativas em dias e turnos diferentes. Por fim, a amostra contou com 58 participantes, sendo alocados 29 no GI e 29 no GC. O diagrama do estudo, de acordo com o CONSORT, é apresentado na Figura 1.

**Figura 1 -:** Diagrama do estudo de acordo com *Consolidated Standards of Reporting Trials* (CONSORT)

As características sociodemográficas, condição de saúde e situação como cuidador no GI e GC são apresentadas na Tabela 1. Os grupos diferiram estatisticamente apenas em relação ao parentesco (p=0,034), observada maior proporção de filhos no GC. As características sociodemográficas e condições de saúde dos idosos sobreviventes de AVC foram semelhantes entre os grupos e são apresentadas na Tabela 2. Ressalta-se que apesar de não haver diferença significativa entre os grupos em relação à MIF, o escore total aumentou em ambos, quando comparadas às avaliações basal e final, sendo esta diferença intragrupo estatisticamente significativa (p<0,001).

**Tabela 1 -:** Características sociodemográficas, condição de saúde e situação como cuidador em relação aos cuidadores familiares (n = 58). Porto Alegre, RS, Brasil, 2024

**Variáveis**	GI* **(n=29)**	**GC** ^†^ **(n=29)**	**p**
Idade (anos)^‡^	52,1 ± 13,3	51,4 ± 11,6	0,818
Sexo - Feminino^§^	22 (75,9)	24 (82,8)	0,746
Situação conjugal^§^ - Com companheiro	18 (62,1)	14 (48,3)	0,428
*Status* Profissional^§^ - Com ocupação	15 (51,7)	20 (69,0)	0,283
Escolaridade^§^			0,840
Fundamental	5 (17,2)	6 (20,7)	
Médio	14 (48,3)	15 (51,7)	
Superior/Pós-graduação	10 (34,5)	8(27,6)	
Parentesco^§^			0,034
Filho	13 (44,8)	18 (62,1)	
Cônjuge	6 (20,7)	9 (31,0)	
Outros familiares	10 (34,5)	2 (6,9)	
Problemas de saúde^§^	15 (51,7)	18 (62,1)	0,596
Doenças cardiovasculares	9 (31,0)	14 (48,3)	0,283
Diabetes *Mellitus*	2 (6,9)	3 (10,3)	1,000
Renda familiar^||^ (salário-mínimo)^¶^	3000 (2500-5000)	2640 (1950-3950)	0,180
Reside com o idoso (sim)^§^	20 (69,0)	18 (62,1)	0,550
Tempo que reside com o idoso (anos)^||^	20 (5 – 45)	30 (10 – 43)	0,361
Dias de cuidado semanal^‡^	6,0 ± 1,7	6,1 ± 1,8	0,878
Experiência como cuidador (sim)^§^	20 (69,0)	22 (75,9)	0,769
Recebe ajuda para o cuidado (sim)^§^	17 (58,6)	12 (41,4)	0,294
Tipo de ajuda^§^			
Instrumental	16 (94,1)	8 (66,7)	0,130
Emocional	6 (35,3)	5 (41,7)	1,000
Financeira	10 (58,8)	4 (33,3)	0,329
Já recebeu treinamento para cuidar de alguém^§^	1 (3,4)	6 (20,7)	0,102

*GI = Grupo intervenção; ^†^GC = Grupo controle; ^‡^Média ± desvio-padrão; ^§^Número absoluto e (%); ^||^Mediana e percentis 25 e 75; ^¶^O salário-mínimo na coleta de dados foi de R$ 1.320,00, Brasil, 2023

**Tabela 2 -:** Características sociodemográficas e condição de saúde dos idosos sobreviventes de AVC* (n = 58). Porto Alegre, RS, Brasil, 2024

**Variáveis**	**GI** ^†^ **(n=29)**	**GC** ^‡^ **(n=29)**	**p**
Idade (anos)^§^	72,9 ± 7,0	71,8 ± 7,3	0,572
Sexo Feminino^||^	18 (62,1)	13 (44,8)	0,292
Estado conjugal^||^ - Com companheiro	14 (48,3)	17 (58,6)	0,599
*Status* profissional^||^ - Sem ocupação	28 (96,6)	27 (93,1)	1,000
Escolaridade^||^			0,180
Fundamental	17 (58,6)	17 (58,6)	
Médio	9 (31,0)	12 (41,4)	
Superior/Pós-graduação	3 (10,3)	0 (0,0)	
AVC* – Isquêmico^||^	26 (89,7)	26 (89,7)	1,000
Morbidades^||^	28 (96,6)	28 (96,6)	1,000
Doenças cardiovasculares	26 (89,7)	25 (86,2)	1,000
Diabete *Mellitus*	7 (24,1)	10 (34,5)	0,564
Local de Internação^||^			0,218
UCE^¶^-AVC*	23 (79,3)	19 (65,5)	
Emergência	5 (17,2)	10 (34,4)	
Unidade de Internação	1 (3,4)	0 (0,0)	
Capacidade Funcional Total da MIF**			
Basal	62,7 ± 31,2	60,0 ± 28,9	0,829^††^
Final	101,8 ± 19,8	90,4 ± 26,7	0,144^††^
Diferença	39,1 (28,2 a 50,1)	30,4 (20,9 a 39,9)	0,472^††^
O idoso teve alguma reinternação hospitalar após a alta^||^	3 (12,0)	5 (20,0)	0,702
O idoso precisou de atendimento de emergência^||^	6 (24,0)	6 (24,0)	1,000

*AVC = Acidente Vascular Cerebral; ^†^GI = Grupo intervenção; ^‡^GC = Grupo controle; ^§^Média ± desvio-padrão; ^||^Número absoluto e (%); ^¶^UCE = Unidade de Cuidados Especiais; **MIF = Medida da Independência Funcional; ^††^Efeito da intervenção entre os grupos pelo modelo de Equações de Estimativas Generalizadas (GEE) complementado pelo teste *Least Significant Difference* (LSD), com valor de p ajustado para o parentesco

Os efeitos da intervenção nas pontuações da escala ECCIID- AVC são apresentados na Tabela 3. Foi observado o aumento estatisticamente significativo da pontuação total da escala em ambos os grupos (p<0,001). No entanto, na comparação intergrupo, não houve diferença estatisticamente significativa (p=0,604). Todavia, na comparação dos itens da escala ECCIID-AVC entre os grupos, observaram-se escores significativamente mais elevados na questão “Ajuda na administração dos medicamentos conforme a prescrição médica” no GI (p=0,006), ao final da intervenção.

Na comparação dos itens da escala intragrupo houve aumento estatisticamente significativo em ambos os grupos, nas questões: Coloca os alimentos e utensílios no lado em que o idoso apresenta maior dependência para estimular o membro afetado; Fornece apoio e/ou materiais necessários para facilitar a alimentação; Fornece apoio e/ou materiais necessários para facilitar a higiene pessoal; Providencia a privacidade durante o uso do sanitário, na troca de fraldas ou no banho; Fornece apoio e/ou materiais necessários para facilitar o vestir; Explica ao idoso qual é a maneira certa para se transferir de lugar; Fornece apoio e/ou materiais necessários para o idoso se transferir de lugar; Fornece apoio e/ou materiais necessários para posicionar o idoso.

Somente no GI houve aumento estatisticamente significativo nas questões: Controla a ingestão de alimentos; Ajuda na administração dos medicamentos conforme a prescrição médica; Ajuda o idoso a se transferir de lugar; Avalia a necessidade de revezar a posição do corpo do idoso. Por outro lado, neste grupo houve redução estatisticamente significativa do item: Mantém uma aparência bem cuidada.

Além do mais, houve aumento estatisticamente significativo somente no GC dos itens: Introduz água, caso a sonda fique obstruída durante a administração da dieta e de medicamentos; Introduz água para lavagem da sonda após a administração da dieta e de medicamentos; Fornece apoio e/ou materiais necessários para facilitar as eliminações urinárias e intestinais; Ajuda na higiene íntima após o uso do sanitário ou troca de fraldas; Avalia a capacidade do idoso para se transferir de lugar.

Observa-se uma melhora similar na capacitação dos cuidadores de ambos os grupos. Não foram observadas alterações estatisticamente significativas nos escores de capacitação das outras questões.

**Tabela 3 -:** Efeitos da intervenção nos escores da ECCIID-AVC* dos cuidadores familiares (n = 58). Porto Alegre, RS, Brasil, 2024

**Itens**	**GI** ^†^ **Média ± DP** ^||^	**GC** ^‡^ **Média ± DP** ^||^	**p**_ajustado_^§^
Total			
Basal	2,34 ± 0,44	2,34 ± 0,41	0,812
Final	2,66 ± 0,31	2,72 ± 0,19	0,269
Diferença	0,31 (0,15 a 0,48)	0,38 (0,19 a 0,57)	0,604
p	<0,001	<0,001	
Prepara as refeições de acordo com a dieta prescrita ou orientada.			
Basal	2,45 ± 0,87	2,69 ± 0,60	0,065
Final	2,68 ± 0,65	2,82 ± 0,39	0,225
Diferença	0,23 (-0,11 a 0,58)	0,13 (-0,10 a 0,36)	0,405
p	0,187	0,268	
Prepara a refeição de uma forma adequada.			
Basal	2,48 ± 0,79	2,62 ± 0,56	0,365
Final	2,74 ± 0,54	2,86 ± 0,47	0,581
Diferença	0,26 (-0,12 a 0,63)	0,24 (-0,06 a 0,54)	0,944
p	0,181	0,112	
Coloca os alimentos e utensílios no lado em que o idoso apresenta maior dependência para estimular o membro afetado.			
Basal	1,61 ± 1,32	1,41 ± 1,09	0,913
Final	2,24 ± 1,09	2,14 ± 1,24	0,781
Diferença	0,63 (0,02 a 1,24)	0,73 (0,23 a 1,23)	0,766
p	0,042	0,004	
Fornece apoio e/ou materiais necessários para facilitar a alimentação.			
Basal	2,56 ± 0,64	2,57 ± 0,63	0,906
Final	2,96 ± 0,20	2,96 ± 0,20	0,979
Diferença	0,40 (0,17 a 0,64)	0,39 (0,15 a 0,63)	0,913
p	<0,001	0,002	
Controla a ingestão de alimentos.			
Basal	2,41 ± 1,02	2,59 ± 0,84	0,327
Final	2,87 ± 0,34	2,90 ± 0,31	0,366
Diferença	0,46 (0,09 a 0,83)	0,31 (-0,03 a 0,64)	0,547
p	0,016	0,074	
Vigia a deglutição.			
Basal	2,50 ± 1,00	2,63 ± 0,74	0,444
Final	2,79 ± 0,42	2,89 ± 0,32	0,176
Diferença	0,29 (-0,09 a 0,67)	0,27 (-0,06 a 0,59)	0,906
p	0,132	0,110	
Ajuda na administração dos medicamentos conforme a prescrição médica.			
Basal	2,66 ± 0,72	2,79 ± 0,49	0,537
Final	3,00 ± 0,00	2,78 ± 0,42	0,006
Diferença	0,34 (0,09 a 0,60)	-0,01 (-0,28 a 0,26)	0,074
p	0,009	0,940	
Introduz água, caso a sonda fique obstruída durante a administração da dieta e de medicamentos.			
Basal	0,60 ± 0,89	0,20 ± 0,45	0,252
Final	2,00 ± 1,73	3,00 ± 0,00	0,364
Diferença	1,40 (-0,23 a 3,03)	2,80 (2,45 a 3,15)	0,200
p	0,091	<0,001	
Introduz água para lavagem da sonda após a administração da dieta e de medicamentos.			
Basal	0,60 ± 0,89	0,20 ± 0,45	0,252
Final	2,00 ± 1,73	3,00 ± 0,00	0,364
Diferença	1,40 (-0,23 a 3,03)	2,80 (2,45 a 3,15)	0,200
p	0,091	<0,001	
Realiza a hidratação da pele.			
Basal	2,39 ± 0,96	2,39 ± 0,92	0,783
Final	2,41 ± 0,87	2,65 ± 0,70	0,288
Diferença	0,02 (-0,56 a 0,60)	0,25 (-0,11 a 0,62)	0,507
p	0,949	0,176	
Prepara o material de higiene.			
Basal	2,72 ± 0,53	2,61 ± 0,74	0,646
Final	2,61 ± 0,85	2,86 ± 0,35	0,202
Diferença	-0,11 (-0,52 a 0,29)	0,26 (-0,07 a 0,58)	0,164
p	0,586	0,118	
Fornece apoio e/ou materiais necessários para facilitar a higiene pessoal.			
Basal	2,72 ± 0,53	2,45 ± 0,91	0,110
Final	3,00 ± 0,00	2,96 ± 0,21	0,177
Diferença	0,28 (0,09 a 0,46)	0,51 (0,16 a 0,85)	0,222
p	0,004	0,004	
Ajuda no banho.			
Basal	2,52 ± 0,74	2,41 ± 0,80	0,463
Final	2,50 ± 0,91	2,68 ± 0,48	0,626
Diferença	-0,02 (-0,59 a 0,56)	0,28 (-0,09 a 0,65)	0,417
p	0,953	0,141	
Ajuda na higiene oral.			
Basal	2,55 ± 0,78	2,69 ± 0,55	0,676
Final	1,86 ± 1,35	2,86 ± 0,36	0,065
Diferença	-0,69 (-1,67 a 0,28)	0,16 (-0,14 a 0,47)	0,168
p	0,162	0,296	
Mantém uma aparência bem cuidada.			
Basal	2,93 ± 0,26	2,69 ± 0,71	0,048
Final	2,67 ± 0,58	2,75 ± 0,55	0,743
Diferença	-0,26 (-0,51 a -0,01)	0,06 (-0,31 a 0,43)	0,164
p	0,038	0,752	
Providencia a privacidade durante o uso do sanitário, na troca de fraldas ou no banho.			
Basal	2,46 ± 0,84	2,54 ± 0,79	0,532
Final	2,82 ± 0,73	2,88 ± 0,33	0,546
Diferença	0,36 (0,06 a 0,66)	0,35 (0,01 a 0,69)	0,966
p	0,018	0,046	
Fornece apoio e/ou materiais necessários para facilitar as eliminações urinárias e intestinais.			
Basal	2,48 ± 0,80	2,30 ± 0,72	0,473
Final	2,79 ± 0,71	2,95 ± 0,22	0,337
Diferença	0,31 (-0,10 a 0,71)	0,65 (0,35 a 0,95)	0,199
p	0,138	<0,001	
Ajuda na higiene íntima após o uso do sanitário ou troca de fraldas.			
Basal	2,22 ± 0,97	2,20 ± 0,91	0,751
Final	2,25 ± 1,17	2,85 ± 0,38	0,395
Diferença	0,03 (-0,74 a 0,79)	0,65 (0,25 a 1,05)	0,297
p	0,943	0,002	
Fornece apoio e/ou materiais necessários para facilitar o vestir.			
Basal	2,57 ± 0,69	2,69 ± 0,54	0,561
Final	3,00 ± 0,00	2,91 ± 0,29	0,178
Diferença	0,43 (0,18 a 0,68)	0,22 (0,01 a 0,43)	0,254
p	<0,001	0,037	
Ajuda a pessoa a vestir-se.			
Basal	2,46 ± 0,79	2,70 ± 0,54	0,153
Final	2,72 ± 0,46	2,71 ± 0,46	0,811
Diferença	0,26 (-0,06 a 0,58)	0,01 (-0,28 a 0,30)	0,289
p	0,115	0,942	
Avalia a capacidade do idoso para se transferir de lugar.			
Basal	2,28 ± 0,99	2,36 ± 0,95	0,724
Final	2,67 ± 0,70	2,82 ± 0,50	0,311
Diferença	0,39 (-0,07 a 0,85)	0,46 (0,06 a 0,86)	0,774
p	0,095	0,025	
Explica ao idoso qual é a maneira certa para se transferir de lugar.			
Basal	2,24 ± 0,91	2,00 ± 1,07	0,639
Final	2,71 ± 0,69	2,65 ± 0,57	0,849
Diferença	0,47 (0,09 a 0,84)	0,65 (0,21 a 1,10)	0,594
p	0,015	0,004	
Fornece apoio e/ou materiais necessários para o idoso se transferir de lugar.			
Basal	2,17 ± 0,97	2,03 ± 0,94	0,729
Final	2,86 ± 0,64	2,95 ± 0,22	0,403
Diferença	0,69 (0,37 a 1,02)	0,92 (0,55 a 1,28)	0,344
p	<0,001	<0,001	
Ajuda o idoso a se transferir de lugar.			
Basal	2,21 ± 0,98	2,39 ± 0,74	0,268
Final	2,67 ± 0,73	2,76 ± 0,56	0,504
Diferença	0,46 (0,10 a 0,82)	0,37 (-0,05 a 0,80)	0,658
p	0,011	0,085	
Utiliza postura adequada para transferir o idoso de lugar.			
Basal	1,61 ± 1,07	1,68 ± 0,98	0,481
Final	1,95 ± 1,00	2,00 ± 0,86	0,659
Diferença	0,35 (-0,11 a 0,81)	0,32 (-0,11 a 0,75)	0,766
p	0,138	0,142	
Fornece apoio e/ou materiais necessários para posicionar o idoso.			
Basal	2,15 ± 0,91	2,36 ± 0,91	0,398
Final	2,94 ± 0,24	2,80 ± 0,52	0,300
Diferença	0,80 (0,48 a 1,12)	0,44 (0,02 a 0,86)	0,222
p	<0,001	0,040	
Avalia a necessidade de revezar a posição do corpo do idoso.			
Basal	2,29 ± 0,90	2,31 ± 0,85	0,961
Final	2,65 ± 0,61	2,43 ± 0,65	0,275
Diferença	0,36 (0,05 a 0,67)	0,12 (-0,32 a 0,55)	0,353
p	0,022	0,593	
Utiliza postura adequada para posicionar cada parte do corpo do idoso corretamente.			
Basal	1,74 ± 1,13	1,61 ± 1,07	0,646
Final	2,17 ± 0,79	1,95 ± 0,83	0,395
Diferença	0,43 (-0,12 a 0,98)	0,34 (-0,11 a 0,80)	0,898
p	0,129	0,138	
Reveza a posição do corpo do idoso quando ele está deitado.			
Basal	2,16 ± 1,07	2,13 ± 0,95	0,901
Final	2,57 ± 0,79	2,11 ± 0,93	0,251
Diferença	0,41 (-0,31 a 1,13)	-0,01 (-0,75 a 0,72)	0,417
p	0,262	0,970	

*ECCIID–AVC = Escala de Capacidades do Cuidador Informal de Idosos Dependentes por Acidente Vascular Cerebral; ^†^GI = Grupo intervenção; ^‡^GC = Grupo controle; ^§^Efeito da intervenção entre os grupos pelo modelo de Equações de Estimativas Generalizadas (GEE), complementado pelo teste *Least Significant Difference* (LSD) e com valor de p ajustado para o parentesco; ^¶^Média ± desvio-padrão

Em relação à sobrecarga do cuidador familiar, o efeito da intervenção está apresentado na Tabela 4. Não houve diferença significativa entre os grupos. Em relação à sobrecarga total, observou-se uma redução estatisticamente significativa em ambos os grupos (p<0,001). Nas comparações dos domínios intragrupo, houve redução estatisticamente significativa nos domínios tensão geral, isolamento e ambiente em ambos os grupos. No domínio decepção houve redução significativa apenas no GI (p=0,011).

**Tabela 4 -:** Efeitos da intervenção nos escores da CBS* dos cuidadores familiares (n = 58). Porto Alegre, RS, Brasil, 2024

**Domínios**	**GI** ^†^ **Média ± DP** ^||^	**GC** ^‡^ **Média ± DP** ^||^	**p**_ajustado_^§^
CBS* total			
Basal	1,87 ± 0,46	2,01 ± 0,51	0,570
Final	1,53 ± 0,43	1,60 ± 0,51	0,912
Diferença	-0,34 (-0,52 a -0,17)	-0,41 (-0,64 a -0,18)	0,679
p	<0,001	<0,001	
Tensão geral			
Basal	2,02 ± 0,65	2,12 ± 0,76	0,916
Final	1,69 ± 0,68	1,64 ± 0,69	0,533
Diferença	-0,33 (-0,64 a -0,03)	-0,48 (-0,82 a -0,14)	0,542
p	0,033	0,006	
Isolamento			
Basal	1,67 ± 0,62	2,06 ± 1,01	0,099
Final	1,24 ± 0,43	1,46 ± 0,74	0,296
Diferença	-0,43 (-0,71 a -0,15)	-0,60 (-0,94 a -0,26)	0,443
p	0,003	<0,001	
Decepção			
Basal	1,90 ± 0,75	1,83 ± 0,62	0,417
Final	1,54 ± 0,47	1,57 ± 0,61	0,841
Diferença	-0,36 (-0,64 a -0,08)	-0,26 (-0,54 a 0,02)	0,555
p	0,011	0,069	
Envolvimento emocional			
Basal	1,14 ± 0,24	1,27 ± 0,63	0,462
Final	1,20 ± 0,38	1,16 ± 0,36	0,467
Diferença	0,06 (-0,04 a 0,16)	-0,11 (-0,37 a 0,15)	0,227
p	0,254	0,392	
Ambiente			
Basal	2,26 ± 0,63	2,50 ± 0,79	0,409
Final	1,70 ± 0,57	1,94 ± 0,72	0,350
Diferença	-0,56 (-0,84 a -0,28)	-0,56 (-0,90 a -0,21)	0,974
p	<0,001	0,001	

*CBS = *Caregiver Burden Scale*; ^†^GI = Grupo intervenção; ^‡^GC = Grupo controle; ^§^Efeito da intervenção entre os grupos pelo modelo de Equações de Estimativas Generalizadas (GEE), complementado pelo teste *Least Significant Difference* (LSD) e com valor de p ajustado para o parentesco; ^||^Média ± desvio-padrão

Quanto aos dados de acesso ao curso, a média do total de acessos foi de 10,3 (± 5,4), já a média do número de módulos acessados foi de 7,8 (± 2,5). Não houve interferência estatisticamente significativa do total de acessos (p=0,164) e do número de módulos acessados (p=0,182) na mudança dos escores da ECCIID-AVC no GI. O módulo mais acessado foi o Módulo 1 - O que é AVC (36 acessos), seguido pelo Módulo 4 - Cuidados com medicamentos, (32 acessos). O menos acessado foi o Módulo 11 - Cuidados com traqueostomia (14 acessos).

## Discussão

Este EPR analisou os efeitos de uma intervenção educativa virtual direcionada a cuidadores familiares de idosos sobreviventes de AVC. Foi observada uma similaridade entre os dois grupos, no que diz respeito à melhora da capacitação e à redução da sobrecarga dos cuidadores. O maior resultado deste estudo afirma que a intervenção favoreceu significativamente os cuidadores que a receberam em relação à melhora da capacidade para administração de medicamentos.

A respeito da caracterização dos participantes, a maioria dos cuidadores eram mulheres, com média de idade em torno dos 50 anos e maior proporção de filhos no GC quando comparado ao GI, o que chama atenção pois, no Brasil, o papel de cuidar da pessoa idosa dependente é historicamente direcionado às filhas e às esposas/companheiras^(24)^. Este dado pode ter interferido nos resultados obtidos, na medida em que os filhos assumem a responsabilidade pelo cuidado porque se sentem na obrigação, porém o sentimento de dever pode transformar-se em motivação e inspiração para cuidar dos pais idosos, apesar das dificuldades em se adaptar a esse novo papel^(25)^.

Ainda, com relação às características dos participantes, observou-se que havia no GC mais cuidadores que receberam treinamento formal para cuidar previamente ao estudo. Além disso, neste grupo, 75,9% dos participantes já haviam cuidado de outra pessoa dependente, a mesma situação foi observada em 69% dos cuidadores do GI. Esses dados indicam que, possivelmente, os cuidadores estudados já haviam desenvolvido alguma autoconfiança sobre sua competência, conhecimento e habilidades para realizar tarefas diárias e lidar com suas emoções^(26)^, sofrendo menor interferência da intervenção.

Quanto às características dos idosos, as médias da escala MIF indicaram que os sobreviventes ao AVC estavam menos dependentes, na avaliação final para os dois grupos, apesar de não haver significância estatística. Tal fato pode ser justificado considerando que a maioria dos idosos teve um evento isquêmico, que tende a gerar menor dependência ao longo do tempo quando comparado ao hemorrágico^(27)^.

Quando analisado cada item da escala ECCIID-AVC, observa-se uma melhora em quatro itens para o GI e em cinco itens para o GC, indicando incremento similar na capacitação dos cuidadores, o que pode ser creditado à própria experiência de cuidado. No item sobre administração de medicamentos, os cuidadores do GI tiveram melhores resultados ao final da intervenção, mesmo que a mudança entre os grupos não tenha sido significativa. Além disso, o módulo sobre os cuidados com medicamentos foi o mais acessado pelos cuidadores, ficando atrás apenas do módulo introdutório sobre o que é o AVC. Estudo com cuidadores familiares de idosos dependentes identificou que o cuidado com as medicações representou uma das principais atividades com dificuldade de ser realizada, o que ocorre pela falta de informação e de conhecimento para seu desempenho^(28)^.

O estudo português que desenvolveu o instrumento original Escala de Capacidades do Prestador Informal de Cuidados de Idosos Dependentes por AVC (ECPICID-AVC), validado posteriormente para uso no Brasil como ECCIID-AVC^(19)^, aplicou um programa de treinamento chamado *Intervention in Informal Caregivers who take Care of Older People after a Stroke* (*InCARE*), em que 85 cuidadores familiares de idosos que sobreviveram ao AVC receberam orientações visando o aumento do conhecimento e das habilidades práticas, por meio de visitas domiciliares (VDs) e telefonemas, durante três meses após a alta. O controle (n = 89) recebeu o cuidado habitual de saúde. Por conseguinte, os resultados indicaram que o grupo experimental obteve maior pontuação média da ECPICID-AVC ao longo do tempo e diminuição de sobrecarga^(29)^. Até o momento, não foram encontrados outros estudos que utilizaram a ECCIID-AVC para avaliação da capacitação dos cuidadores após uma intervenção.

No entanto, identificam-se na literatura diversos estudos de intervenção dedicados à melhoria da capacitação de cuidadores familiares de pessoas dependentes por AVC. Ensaio clínico randomizado (ECR) chinês avaliou os efeitos de uma intervenção psicoeducacional (*Family-focused dyadic psychoeducational intervention* – FDPEI) na sobrecarga e na competência do cuidador. A fase pré-alta englobou três sessões presenciais e visou ao preparo para a transição do hospital para a casa. A fase pós-alta incluiu quatro telefonemas semanais de aconselhamento, encorajando os cuidadores a identificarem dificuldades e motivá-los a lidar com elas. Observou-se redução significativa na sobrecarga e melhora na competência do cuidador para cuidar^(30)^.

Estudo tailandês também ofereceu intervenção, feita por enfermeiros, que consistia em sessões visando aumentar as habilidades comportamentais dos cuidadores com base em informação e motivação. Com duração de oito semanas, a intervenção incluiu uma fase pré-alta, de orientações sobre o AVC, e 2 VDs pós-alta. Concluiu-se que a intervenção melhorou as habilidades de cuidados dos cuidadores familiares^(31)^.

Ao contrário da intervenção *e-share*, o programa FDPEI e o estudo tailandês realizaram múltiplas sessões presenciais pré-alta. Sabe-se que para que o cuidado domiciliar seja realizado de maneira que supra as necessidades do idoso com AVC e reduza os impactos no cuidador, ele deve começar a ser preparado e planejado antes da alta hospitalar^(8)^. A realização de apenas uma sessão presencial antes da alta pode não ter sido suficiente para obtenção de melhores resultados neste estudo.

Estudo realizado em Hong Kong aplicou intervenção realizada por equipe multidisciplinar através de um programa educativo de 26 semanas. O grupo de intervenção demonstrou melhorias significativas em termos de competência de cuidado e um menor nível de sobrecarga três meses após a intervenção^(32)^. Outro estudo, realizado no Peru, avaliou o efeito de oito vídeos educacionais com temas sobre posicionamento, mobilização e transferências, elaborado por equipe multiprofissional, para melhorar as habilidades práticas e o conhecimento de dez cuidadores informais de pacientes com AVC. A pontuação de habilidades práticas e de conhecimento aumentou significativamente^(33)^.

As pesquisas realizadas em Hong Kong e no Peru ofereceram uma combinação de estratégias de cuidado por meio de uma equipe multidisciplinar. Essas diferentes abordagens podem explicar a ausência de diferenças nos escores de capacitação e de sobrecarga dos cuidadores do *e-share*. No entanto, quanto ao estudo realizado no Peru, destaca-se a limitação dos pesquisadores por não terem avaliado os cuidadores após a experiência do cuidado e a amostra reduzida.

No que se refere à sobrecarga enfrentada pelos cuidadores familiares de idosos sobreviventes de AVC, está bem documentada na literatura, especialmente no que diz respeito à transição para o domicílio^(34)^. Logo, espera-se que cuidadores que são melhor capacitados experimentarão menor sobrecarga. Nesse sentido, um estudo nigeriano avaliou o papel moderador da preparação para o cuidado na sobrecarga de cuidadores familiares. Os resultados mostraram que a preparação moderou a relação entre sobrecarga, saúde física e mental dos cuidadores^(35)^.

Garantir que os cuidadores familiares estejam adequadamente preparados e tenham apoio pode reduzir os impactos negativos do cuidado^(35)^. Entre os cuidadores do *e-share*, observou-se redução da sobrecarga total em ambos os grupos. Além do mais, apesar de não haver efeito da intervenção entre grupos, houve redução significativa no domínio “Decepção” apenas no GI, o que pode ser explicado pelo fato de que a intervenção ofereceu disponibilidade de contato, reforçou a importância do envolvimento de todos os membros da família no cuidado e de que o cuidador principal precisava de momentos de descanso, informações contidas no MOOC no Módulo 2.

Apesar de não ter constatado interferência significativa dos acessos ao MOOC e mudança dos escores da ECCIID- AVC, percebe-se coerência entre os módulos mais acessados e as necessidades de cuidado identificadas.

Um estudo piloto de ECR, realizado por internet e telefone nos Estados Unidos, avaliou a viabilidade e aceitabilidade de uma intervenção de suporte e de solução de problemas para cuidadores de sobreviventes de AVC. A intervenção de dois braços, ministrada por enfermeiros, utilizou como base o *siteRESCUE*, que inclui as seções: (1) fichas informativas; (2) lista de recursos adicionais; (3) autogestão; (4) glossário de termos relacionados ao AVC; (5) depoimentos; (6) módulo de treinamento; e (7) diário de resolução de problemas. A intervenção foi realizada por telefone, em quatro ou oito sessões semanais, com duração de 30-60 minutos cada, adaptada aos problemas de cada cuidador, durante oito semanas após a alta hospitalar. Os resultados indicaram respostas positivas à intervenção; paralelamente, a análise qualitativa revelou que a intervenção foi valorizada e aceitável pelos cuidadores^(36)^.

Chama a atenção que o estudo supracitado, com efeitos significativos, foi desenvolvido em país com condições sociais e econômicas mais favoráveis, diferindo das características encontradas no Brasil. Ainda assim, destaca-se que o uso dessas modalidades contribui para a universalização do acesso digital e da informação em saúde.

As intervenções educativas para cuidadores, nos últimos 15 anos, têm sido realizadas em ambientes reais, e com a pandemia de COVID-2019, as pesquisas em ambientes virtuais foram intensificadas. Uma revisão sistemática com metanálise buscou elucidar as evidências relacionadas à utilização de *e-Health* (ferramentas e soluções digitais e/ou virtuais destinadas à saúde) na melhoria da QV de cuidadores informais de pacientes dependentes por AVC. A análise concluiu que houve uma tendência de melhora na saúde mental dos cuidadores; melhor capacidade para a resolução de problemas ligados ao cuidado; e a prevenção dos problemas decorrentes da sobrecarga^(10)^.

Este EPR tem limitações. A maioria dos idosos sobreviventes ao AVC foram captados na UCE-AVC (23 GI *vs.* 19 GC), local onde os cuidadores também contavam com o apoio de profissionais de saúde com conhecimentos altamente especializados. Este fato, associado à característica da amostra de que os participantes de ambos os grupos tinham experiência prévia como cuidadores, pode ter contribuído para encontrarmos resultados não significativos. Para um estudo futuro, é necessária uma amostra mais inclusiva de idosos de instituições não especializadas.

Infere-se que a avaliação dos desfechos em um tempo de seguimento maior também possa interferir nos resultados, uma vez que a própria experiência do cuidado pode ter favorecido a similaridade de resultados entre os grupos. No Brasil, enfermeiras conduziram o ECR *Nursing Home Care Intervention Post Stroke* (SHARE), que consistiu no preparo dos cuidadores para realização das AVDs dos idosos que sofreram AVC, após a alta. Os resultados demonstraram QV semelhante e aumento na sobrecarga total no GC e GI. No entanto, no que diz respeito à QV, cuidadores do GI apresentaram escores de relações sociais mais elevados. Por sua vez, o GI exibiu menor isolamento em relação à sobrecarga, e a avaliação de desfechos ocorreu em 60 dias e um ano após a alta hospitalar^(37-38)^.

Ainda, destaca-se a ausência de pesquisas para fins de comparação das alterações nos escores da ECCIID-AVC como uma limitação. Esperamos que este estudo estimule pesquisadores a adotarem o seu uso, bem como a expansão do uso de tecnologias virtuais, em investigações brasileiras.

Para além da pesquisa, este estudo tem aplicação prática: os dados serão divulgados para o serviço de saúde e o MOOC ficará disponível em plataforma aberta para os cuidadores do GC e para a população. Espera-se, desta forma, apoiar cuidadores familiares que não têm acesso aos serviços de saúde com equipe especializada.

Este é o primeiro estudo no Brasil que propõe o uso do MOOC como ferramenta educativa para cuidadores familiares de idosos sobreviventes de AVC, representando um importante avanço da Enfermagem na construção de tecnologias educacionais digitais.

## Conclusão

A intervenção multicomponente, *e-share*, disponibilizou, além do MOOC, uma linha telefônica e quatro contatos programados. Ainda assim, não se observou efeito da intervenção na melhora da capacitação e na redução da sobrecarga dos cuidadores. Contudo, favoreceu significativamente os cuidadores do GI no que se refere à melhora da administração de medicamentos e à sobrecarga no domínio decepção.

Em suma, este estudo revela a complexidade das ações de transição do cuidado após um AVC. O uso de tecnologias virtuais simples explora alternativas para essa transição que forneçam suporte aos cuidadores para aquisição de habilidades e conhecimentos. Mostra-se necessário que os cuidadores sejam avaliados antes da alta hospitalar do idoso, garantindo uma adequada transição e reduzindo os efeitos negativos do cuidado, destacando o papel central do enfermeiro nessas ações.

# Translation: Efectividad de la intervención *e-share* para cuidadores de personas mayores después de accidente cerebrovascular: ensayo pragmático aleatorizado*

**DOI:** 10.1590/1518-8345.7414.4466

**Authors:** Francine Melo da Costa, Débora Francisco do Canto, Lisiane Manganelli Girardi Paskulin

**Affiliations:** 1 Universidade Federal do Rio Grande do Sul, Escola de Enfermagem, Porto Alegre, RS, Brasil; 2 Hospital de Clínicas de Porto Alegre, Serviço de Enfermagem Ambulatorial, Porto Alegre, RS, Brasil; 3 Hospital de Clínicas de Porto Alegre, Serviço de Enfermagem Clínica, Porto Alegre, RS, Brasil; 4 Hospital de Clínicas de Porto Alegre, Diretoria de Enfermagem, Porto Alegre, RS, Brasil; 5 Becaria del Conselho Nacional de Desenvolvimento Científico e Tecnológico (CNPq), Brasil

**Keywords:** Cuidadores, Educación, Accidente Cerebrovascular, Transición del Hospital al Hogar, Anciano, Tecnología Educacional

## Abstract

**Objetivo:**

analizar la efectividad de una intervención educativa virtual para cuidadores familiares en la sobrecarga y en la capacidad de cuidar a personas mayores después de un accidente cerebrovascular.

**Método:**

ensayo pragmático aleatorizado con 58 cuidadores de personas mayores sobrevivientes de accidente cerebrovascular. El grupo de intervención recibió acceso a un curso en línea para la educación de los cuidadores. La capacitación del cuidador fue evaluada mediante la Escala de Capacidades del Cuidador Informal de Ancianos Dependientes por Accidente Cerebrovascular y la sobrecarga mediante la *Caregiver Burden Scale* antes y tres meses después del alta hospitalaria, ambas adaptadas y validadas para uso en Brasil. Se adoptó el modelo de Ecuaciones de Estimaciones Generalizadas complementado por el *Least Significant Difference*

**Resultados:**

la muestra fue homogénea y los grupos difirieron estadísticamente solo en relación con el parentesco (p=0,034), observándose una mayor proporción de hijos en el grupo control. Hubo un efecto de la intervención en la mejora de la administración de medicamentos (p=0,006) y en la reducción de la decepción (p=0,011) en el grupo de intervención.

**Conclusión:**

la intervención favoreció a los cuidadores que la recibieron en cuanto a la mejora de la administración de medicamentos y la reducción de la decepción. Este es el primer estudio en Brasil que propone el uso de tecnologías educativas digitales para este grupo, representando un importante avance en Enfermería. Registro clinicaltrial.gov: NCT05553340.

## Destacados

**(1)** Primer estudio, en Brasil, que propone un MOOC para cuidadores de personas mayores después de un ACV. **(2)** Avance de la Enfermería en la construcción de tecnologías educativas digitales. **(3)** La *e-share* ayudó a los cuidadores a mejorar la administración de medicamentos. **(4)** La *e-share* ayudó a los cuidadores a reducir la sobrecarga en el dominio de decepción. **(5)** Las herramientas digitales contribuyen al aprovechamiento del trabajo de los enfermeros.

## Introducción

El Accidente Cerebrovascular (ACV) es una condición neurológica que afecta principalmente a las personas mayores, siendo la segunda causa de muerte en Brasil y en el mundo^(1)^. En Brasil, entre enero y octubre de 2023, alrededor de 150 mil personas fueron hospitalizadas por ACV, de las cuales, aproximadamente, 117 mil (79%) tenían 60 años o más, y cerca de 18 mil (28%) fallecieron^(2)^. Además de la alta tasa de mortalidad, el ACV se considera una de las principales causas de discapacidad en la población de personas mayores. Las personas mayores que sufren un ACV tienen más limitaciones para el autocuidado después del alta hospitalaria, necesitando asistencia^(1)^.

El cuidado informal implica numerosas tareas, como apoyo práctico, emocional, financiero y social^(3)^. En Brasil, la disponibilidad de apoyo de redes formales es limitada, recayendo sobre la familia la responsabilidad de la mayor parte de los cuidados^(4)^. Además, el apoyo al cuidador informal no está claramente establecido en las políticas públicas^(5)^, dejando a las familias con la obligación de cuidar, muchas veces sin la información, capacitación y recursos necesarios^(6)^.

Específicamente, en relación con los cuidadores informales de personas mayores dependientes por ACV, un estudio identificó que las principales actividades de cuidado realizadas eran: proporcionar materiales y/o apoyo para alimentarse, vestirse y administrar los medicamentos. Las actividades en las que los cuidadores presentaron mayor dificultad fueron la transferencia y el posicionamiento^(7)^.

Los estudios citados exponen la existencia de brechas en la calidad de la atención brindada a las familias. En contrapartida, las recomendaciones internacionales para la transición del cuidado del hospital al hogar señalan que la educación de los cuidadores familiares debe incluir la capacitación en técnicas de cuidados personales, como alimentación, transferencia y posicionamiento^(8)^.

Estudios internacionales también han demostrado que las intervenciones educativas dirigidas a cuidadores familiares de personas mayores después de un ACV ayudan a reducir la sobrecarga^(9)^, mejoran la capacidad para asistir a la persona mayor en el hogar^(9)^, mejoran la Calidad de Vida (CV) del cuidador^(8)^ y tienen un impacto positivo en la salud física y mental de los sobrevivientes^(8)^.

Sin embargo, aún no hay evidencias, en el contexto nacional y en condiciones más cercanas a las encontradas en la práctica del cuidado, de que una intervención educativa basada en el desarrollo de habilidades y conocimientos pueda mejorar la capacidad de los cuidadores para brindar atención a la persona mayor. Además, las intervenciones virtuales dirigidas a los cuidadores son alternativas recientes que merecen ser mejor evaluadas^(10)^.

La presente investigación se desarrolló considerando la hipótesis de que la intervención educativa virtual, realizada por enfermeros para cuidadores familiares de personas mayores después de un ACV, mejora la capacidad de cuidado, en comparación con el seguimiento habitual. El concepto de “capacidad” adoptado en este estudio proviene de la Teoría Social Cognitiva, en la cual el autor traduce conocimientos, habilidades y competencias como resultado del término “capacidad”^(11 - 12)^.

Este estudio explora alternativas de transición del cuidado, la optimización de los recursos, la mejor experiencia del cuidado y el mejor aprovechamiento del tiempo de trabajo de los enfermeros, así como evidencia y fortalece su papel educador. Se objetivó analizar la efectividad de una intervención educativa virtual para cuidadores familiares, en la sobrecarga y en la capacidad de cuidar a personas mayores después de un accidente cerebrovascular, cuando se compara con las orientaciones habituales de cuidado, en el período de tres meses tras el alta hospitalaria.

## Método

### Diseño

Estudio Pragmático Aleatorizado (EPA), denominado *Educational Intervention With Digital Technology For Family Caregivers* (*e-share*), registrado en clinicaltrial.gov bajo el número NCT05553340. La investigación siguió las recomendaciones de la extensión del *Consolidated Standards of Reporting Trials* (CONSORT)^(13)^.

### Lugar y período

El estudio se desarrolló en el Servicio de Urgencias, en cinco unidades de hospitalización clínica y en la Unidad de Cuidados Especiales de ACV (UCE-ACV) de un hospital universitario de gran tamaño en el Sur de Brasil.

La UCE-ACV atiende a pacientes con diagnóstico de ACV, cuenta con 10 camas y ofrece seguimiento por un equipo multidisciplinario compuesto por médicos, enfermeros, farmacéuticos, nutricionistas, fisioterapeutas, fonoaudiólogos, trabajadores sociales y psicólogos. Al alta, estos profesionales ofrecen a los pacientes y sus familiares información sobre la enfermedad, además de materiales educativos referentes a los cuidados que se realizarán en el domicilio. Dependiendo de la ocupación de estas camas o de la gravedad de los pacientes, estos pueden ser asignados a otras unidades de hospitalización o ser dados de alta directamente desde la emergencia^(14)^.

El reclutamiento se realizó de enero a junio de 2023, y la recolección de datos, de enero a noviembre de 2023.

### Población y muestra

Los participantes del estudio fueron los cuidadores familiares de personas mayores de 60 años o más. El cuidador familiar se entiende como la persona responsable de los cuidados de la persona mayor en el hogar, no remunerada, que puede o no ser un miembro de la familia^(15)^.

Se incluyeron los cuidadores familiares mayores de 18 años que ejercían el papel de principal proveedor de cuidados, no remunerado, a pacientes de 60 años o más (de ambos sexos), con diagnóstico médico de ACV en la hospitalización actual. Se excluyeron los cuidadores familiares que: (a) no tenían acceso a internet; (b) no estuvieran capacitados para acceder a la intervención virtual, verificado por medio del *checklist* de aptitud para acceso y navegación al curso, elaborada para este estudio; (c) no tuvieran línea telefónica para contacto; (d) estuvieran acompañando a personas mayores que fueran transferidos a instituciones de cuidados a largo plazo después del alta; (e) estuvieran acompañando a personas mayores que fallecieran durante la fase de reclutamiento de participantes.

Se calculó el tamaño de la muestra para detectar diferencias en el promedio de la escala utilizada para la evaluación de la capacitación de los cuidadores, entre los grupos de intervención y control, mediante la herramienta *Power and Sample Size for Health Researchers* (*PSS Health*)^(16)^. Se consideró un poder del 80%, un nivel de significancia del 5% y un tamaño de efecto de Cohen de 0,8. Cuanto mayor es el valor de d de Cohen, más significativa es la discrepancia entre los promedios; valores de d de 0,8 representan efectos grandes^(17)^. Este valor fue adoptado, ya que, en el momento de la ejecución de esta investigación, no había estudios previos que aplicaran la escala adoptada para identificar diferencias en los promedios.

La estimación del tamaño total de la muestra alcanzó 52 participantes, 26 en cada grupo. Sumando un 10% para posibles rechazos, el tamaño muestral fue de 58 participantes, 29 en cada grupo. Los participantes elegibles fueron los cuidadores familiares de pacientes de 60 años o más, con diagnóstico médico de ACV, hospitalizados en las unidades de la institución, monitoreados mediante historiales médicos electrónicos disponibles en el *software* institucional durante el período de recolección de datos.

### Variables e instrumentos

El desenlace primario fue la capacidad de los cuidadores informales para cuidar a personas mayores después de un ACV, y el desenlace secundario fue la sobrecarga del cuidador; los participantes fueron evaluados en el momento de la evaluación basal, durante la hospitalización, y en la evaluación final, 90 días después del alta.

Se recolectaron datos sociodemográficos y clínicos de las personas mayores y de sus cuidadores familiares. Para los sobrevivientes de ACV, también se evaluó la Medida de Independencia Funcional (MIF). La MIF evalúa cuantitativamente la carga de cuidados que un paciente dependiente requiere de otra persona para realizar tareas motoras y cognitivas. Está compuesta por 10 categorías divididas en 6 dimensiones: autocuidado; control de esfínteres; transferencias; locomoción; comunicación; y cognición social. Las puntuaciones generales de la MIF pueden variar de 18 a 126. Cuanto menor es la puntuación, mayor es la dependencia de la persona mayor para realizar las tareas. La escala fue validada en el contexto brasileño en 2004, presentando buena correlación en el test/retest y en la reproducibilidad interobservadores (Pearson: 0,91-0,98; coeficiente de correlación intraclase – ICC: 0,91-0,98 y Pearson: 0,87 – 0,98; ICC: 0,87 – 0,98, respectivamente)^(18)^.

El desenlace primario se verificó mediante la Escala de Capacidades del Cuidador Informal de Ancianos Dependientes por ACV (ECCIAD-ACV), que evalúa las diferentes capacidades que los cuidadores familiares tienen o necesitan mejorar en la prestación de cuidados a personas mayores dependientes después de un ACV. Adaptada y validada para uso en Brasil, la ECCIAD-ACV presenta una confiabilidad test-retest satisfactoria (ICC = 0,94; Intervalo de confianza del 95% = 0,91-0,96) y una excelente confiabilidad de consistencia interna (alfa de Cronbach = 0,914)^(19)^.

La escala está compuesta por 29 ítems que abordan cuestiones relacionadas con las siguientes actividades: comer/beber (por vía oral o sonda nasogástrica/gastrostomía); administración de medicamentos; cuidados de higiene (baño y eliminaciones); cuidados de la piel; vestirse/desvestirse; y transferencia/posicionamiento. Estos ítems se evalúan en una escala de 0 (no demuestra - no realiza la actividad) a 3 (demuestra totalmente - realiza la actividad correctamente y de manera autónoma). Además, la opción de respuesta “NA – no se aplica” puede utilizarse en los casos en los que el cuidador no realiza una determinada actividad porque la persona mayor no requiere de ese cuidado^(19)^. La puntuación total de la escala varía de 0 a 87 puntos, sin un punto de corte establecido, y cuanto mayor es la puntuación, más capacitado está el cuidador^(19)^.

El desenlace secundario se evaluó mediante la escala *Caregiver Burden Scale* (CBS)^(20)^, que tiene 22 ítems divididos en 5 dimensiones: tensión general, aislamiento, decepción, implicación emocional y entorno. A las preguntas se les puede asignar respuestas de 1 - de ninguna manera a 4 - frecuentemente. El puntaje total se obtiene mediante la media aritmética de los valores equivalentes a las respuestas de las 22 preguntas, y el puntaje individual se obtiene a partir de la media de los valores equivalentes a las respuestas de las preguntas de cada dimensión. No hay un punto de corte; cuanto mayor sea el puntaje, mayor será la sobrecarga^(20)^. La escala fue adaptada y validada en el contexto brasileño, con coeficientes de reproducibilidad intra e interobservador de 0,87 y 0,92, respectivamente^(20)^.

### Recolección de datos

La recolección de datos comenzó con la identificación de pacientes de 60 años o más, con diagnóstico médico de ACV durante la hospitalización, mediante una consulta diaria al sistema informatizado del hospital. Las personas mayores identificadas recibieron una visita de los investigadores evaluadores para verificar la existencia de un cuidador familiar y si tanto el cuidador como la persona mayor cumplían con los criterios de inclusión del estudio, procediéndose a la aplicación de la lista de verificación de aptitud para acceder al curso. Si el cuidador presente en ese momento no era el cuidador principal, se contactaba por teléfono al cuidador principal para verificar si se cumplían los criterios de inclusión y se programaba la primera evaluación. Aquellos que aceptaron participar firmaron el Consentimiento Informado (CI), y a continuación se recolectaron los datos basales.

El cuidador fue contactado por teléfono tres meses después del alta (90 días) para realizar la evaluación final, por los mismos investigadores responsables de la evaluación inicial. La recolección se realizó mediante videoconferencia por medio de *Google Meet* ^®^ o videollamada por teléfono. El intervalo de tres meses siguió las recomendaciones del estudio de reproducibilidad brasileño de la escala ECCIAD-ACV^(19)^ y cumplió con las evidencias sobre la eficacia de la implementación de programas de intervención educativa, que sugieren al menos tres meses de intervalo entre las evaluaciones^(8)^.

Se realizó una evaluación de la concordancia interevaluadores de las escalas en estudio antes y después del entrenamiento para la recolección de datos. Se adoptó un ICC mayor que 0,70 y un nivel de significancia del 5% (p<0,05), y los análisis se realizaron con el programa *Statistical Package for the Social Sciences* (SPSS), versión 27.0. Después del entrenamiento, todos los ICC fueron mayores de 0,70 y mostraron significancia estadística (p<0,05).

### Aleatorización y enmascaramiento

Después de la aplicación de los instrumentos, los participantes fueron aleatorizados al grupo control (GC) o al grupo intervención (GI). Para ello, se utilizó una lista generada por la página *web* https://www.randomizer.org/, que sigue un orden numérico en el cual cada número ya está asignado a uno de los grupos de manera aleatoria. Los investigadores evaluadores contactaban con un profesional externo al grupo de investigación responsable de la asignación.

Los investigadores que realizaron la evaluación inicial y final y la captación de los participantes estaban cegados para la asignación. En la intervención, se informó al cuidador familiar que recibiría la evaluación final y se le pidió que no mencionara si había recibido la intervención educativa. De la misma manera, se indicó al GC, mediante contacto telefónico por un miembro externo al grupo de investigación, que no informaran al evaluador si no habían recibido la intervención. Las investigadoras intervencionistas solo conocieron a los participantes del GI.

### Intervención

La intervención multicomponente tenía como objetivo capacitar al cuidador familiar para asistir a la persona mayor en las actividades de la vida diaria (AVD) después del alta, y fue realizada por dos enfermeras investigadoras intervencionistas mediante un curso masivo abierto en línea (MOOC). Los detalles sobre la elaboración, construcción y contenido del MOOC se pueden consultar en un artículo previamente publicado^(21)^.

Además de la disponibilidad del MOOC, la intervención incluyó contactos telefónicos con los participantes en los días 7, 30, 60 y 80 después del alta hospitalaria, para verificar el progreso del curso y resolver posibles dificultades. También se puso a disposición una línea directa para los participantes, de lunes a viernes, de 8:00 a 18:00, atendida por las enfermeras, en caso de que hubiera dudas sobre el acceso y la navegación en el curso. La intervención tuvo una duración de tres meses.

Los participantes del GI, después de la asignación, recibieron una visita de instrucción por parte de las investigadoras intervencionistas, aún durante la hospitalización, con el objetivo de proporcionar orientaciones detalladas sobre la intervención. Además, se orientó a los cuidadores sobre los contactos telefónicos y la disponibilidad de la línea directa. En ese momento, se les entregó un folleto con las orientaciones, que también se envió por *WhatsApp* ^®^.

### Control

Para el grupo control, no se ofreció la intervención. El paciente y su cuidador familiar recibieron las orientaciones de cuidado habituales y el seguimiento convencional en la red de servicios a la que tienen acceso (pública o privada), al igual que los participantes del GI.

### Análisis de los datos

Se adoptó la técnica por intención de tratar^(17)^. Para comparar medias, se aplicó la prueba t de Student. En caso de asimetría, se utilizó la prueba de Mann-Whitney. En la comparación de proporciones, se aplicaron las pruebas de chi-cuadrado de Pearson o exacta de Fisher.

Para comparar las escalas entre grupos e intragrupo simultáneamente, se utilizó el modelo de Ecuaciones de Estimaciones Generalizadas (GEE), complementado por la prueba *Least Significant Difference* (LSD). Se aplicó el modelo lineal para las variables numéricas con distribución normal y el modelo logarítmico para aquellas con distribución asimétrica. En el mismo modelo, se ajustó la diferencia de parentesco entre los dos grupos y el número total de accesos y de módulos accedidos para el GI. El nivel de significancia adoptado fue del 5% (p<0,05), y los análisis se realizaron con el programa SPSS, versión 27.0.

### Aspectos éticos

Se observaron las normas de la Resolución nº 466/2012, del *Conselho Nacional de Saúde* ^(22)^, y la Circular nº 2/2021/CONEP/SECNS/MS, sobre investigaciones en entornos virtuales^(23)^. Los participantes firmaron el CI. La investigación fue aprobada por el Comité de Ética en Investigación, Certificado de Presentación para Apreciación Ética (CAAE): 59589922.0.0000.5327, y el protocolo de investigación fue registrado en clinicaltrial.gov bajo la identificación NCT05553340.

## Resultados

Durante el período de reclutamiento, se evaluaron 280 cuidadores para determinar su elegibilidad, de los cuales 222 fueron excluidos, siendo: 129 por no cumplir con el criterio de inclusión de “cuidar a personas mayores con diagnóstico de ACV confirmado en la hospitalización actual”; 15 por cumplir al menos un criterio de exclusión; y 78 por otros motivos: a) nueve por no aceptar participar en el estudio, y b) 69 por no haber podido contactar con el cuidador después de tres intentos en días y turnos diferentes. La muestra incluyó a 58 participantes, siendo asignados 29 al GI y 29 al GC. El diagrama del estudio según CONSORT se presenta en la Figura 1.

**Figura 1 -:** Diagrama del estudio según los *Consolidated Standards of Reporting Trials* (CONSORT)

Las características sociodemográficas, condición de salud y situación como cuidador en el GI y en el GC se presentan en la Tabla 1. Los grupos se diferenciaron estadísticamente solo en relación con el parentesco (p=0,034), observándose una mayor proporción de hijos en el GC. Las características sociodemográficas y las condiciones de salud de las personas mayores sobrevivientes de ACV fueron similares entre los grupos y se presentan en la Tabla 2. Se destaca que, aunque no hubo una diferencia significativa entre los grupos con respecto a la MIF, el puntaje total aumentó en ambos grupos cuando se compararon las evaluaciones basal y final, siendo esta diferencia intragrupo estadísticamente significativa (p<0,001).

**Table:** Tabla 1 Características sociodemográficas, condición de salud y situación como cuidador de los cuidadores familiares (n = 58). Porto Alegre, RS, Brasil, 2024

**Variables**	GI* **(n=29)**	**GC** ^†^ **(n=29)**	**p**
Edad (años)^‡^	52,1 ± 13,3	51,4 ± 11,6	0,818
Sexo - Femenino^§^	22 (75,9)	24 (82,8)	0,746
Estado civil^§^ - Con pareja	18 (62,1)	14 (48,3)	0,428
Situación laboral^§^ - Con empleo	15 (51,7)	20 (69,0)	0,283
Escolaridad^§^			0,840
Primaria	5 (17,2)	6 (20,7)	
Secundaria	14 (48,3)	15 (51,7)	
Superior/Postgrado	10 (34,5)	8(27,6)	
Parentesco^§^			0,034
Hijo	13 (44,8)	18 (62,1)	
Cónyuge	6 (20,7)	9 (31,0)	
Otros familiares	10 (34,5)	2 (6,9)	
Problemas de salud^§^	15 (51,7)	18 (62,1)	0,596
Enfermedades cardiovasculares	9 (31,0)	14 (48,3)	0,283
Diabetes *Mellitus*	2 (6,9)	3 (10,3)	1,000
Ingreso familiar^||^ (salario mínimo)^¶^	3000 (2500-5000)	2640 (1950-3950)	0,180
Reside con la persona mayor (sí)^§^	20 (69,0)	18 (62,1)	0,550
Tiempo de convivencia con la persona mayor (años)^||^	20 (5 – 45)	30 (10 – 43)	0,361
Días de cuidado semanal^‡^	6,0 ± 1,7	6,1 ± 1,8	0,878
Experiencia como cuidador (sí)^§^	20 (69,0)	22 (75,9)	0,769
Recibe ayuda para el cuidado (sí)^§^	17 (58,6)	12 (41,4)	0,294
Tipo de ayuda^§^			
Instrumental	16 (94,1)	8 (66,7)	0,130
Emocional	6 (35,3)	5 (41,7)	1,000
Financiera	10 (58,8)	4 (33,3)	0,329
Ha recibido capacitación para cuidar de alguien^§^	1 (3,4)	6 (20,7)	0,102

*GI = Grupo intervención; ^†^GC = Grupo control; ^‡^Media ± desviación estándar; ^§^Número absoluto y (%); ^||^Mediana y percentiles 25 y 75; ^¶^El salario mínimo durante la recolección de datos fue de R$ 1.320,00, Brasil, 2023

**Table:** Tabla 2 Características sociodemográficas y condición de salud de las personas mayores sobrevivientes de ACV* (n = 58). Porto Alegre, RS, Brasil, 2024

**Variables**	**GI** ^†^ **(n=29)**	**GC** ^‡^ **(n=29)**	**p**
Edad (años)^§^	72,9 ± 7,0	71,8 ± 7,3	0,572
Sexo Femenino^||^	18 (62,1)	13 (44,8)	0,292
Estado civil^|^ - Con pareja	14 (48,3)	17 (58,6)	0,599
Situación laboral^||^ - Sin empleo	28 (96,6)	27 (93,1)	1,000
Escolaridad^||^			0,180
Primaria	17 (58,6)	17 (58,6)	
Secundaria	9 (31,0)	12 (41,4)	
Superior/Postgrado	3 (10,3)	0 (0,0)	
ACV* – Isquémico^||^	26 (89,7)	26 (89,7)	1,000
Morbilidades^||^	28 (96,6)	28 (96,6)	1,000
Enfermedades cardiovasculares	26 (89,7)	25 (86,2)	1,000
Diabetes *Mellitus*	7 (24,1)	10 (34,5)	0,564
Lugar de Hospitalización^||^			0,218
UCE^¶^-ACV*	23 (79,3)	19 (65,5)	
Emergencia	5 (17,2)	10 (34,4)	
Unidad de Hospitalización	1 (3,4)	0 (0,0)	
Capacidad Funcional Total de la MIF**			
Basal	62,7 ± 31,2	60,0 ± 28,9	0,829^††^
Final	101,8 ± 19,8	90,4 ± 26,7	0,144^††^
Diferencia	39,1 (28,2 a 50,1)	30,4 (20,9 a 39,9)	0,472^††^
La persona mayor fue readmitida en el hospital después del alta^||^	3 (12,0)	5 (20,0)	0,702
La persona mayor necesitó atención de emergencia^||^	6 (24,0)	6 (24,0)	1,000

*ACV = Accidente Cerebrovascular; ^†^GI = Grupo intervención; ^‡^GC = Grupo control; ^§^Media ± desviación estándar; ^||^Número absoluto y (%); ^¶^UCE = Unidad de Cuidados Especiales; **MIF = Medida de la Independencia Funcional; ^††^Efecto de la intervención entre los grupos según el modelo de Ecuaciones de Estimaciones Generalizadas (GEE) complementado con la prueba *Least Significant Difference* (LSD), con valor de p ajustado para el parentesco

Los efectos de la intervención en las puntuaciones de la escala ECCIAD-ACV se presentan en la Tabla 3. Se observó un aumento estadísticamente significativo en la puntuación total de la escala en ambos grupos (p<0,001). Sin embargo, en la comparación entre grupos no hubo una diferencia estadísticamente significativa (p=0,604).

En la comparación de los ítems de la escala ECCIAD-ACV entre los grupos, se observaron puntuaciones significativamente más altas en la cuestión “Ayuda en la administración de los medicamentos según la prescripción médica” en el GI (p=0,006), al final de la intervención.

En la comparación de los ítems de la escala intragrupo, hubo un aumento estadísticamente significativo en ambos grupos en las siguientes cuestiones: Coloca los alimentos y utensilios en el lado en que la persona mayor presenta mayor dependencia para estimular el miembro afectado; Proporciona apoyo y/o materiales necesarios para facilitar la alimentación; Proporciona apoyo y/o materiales necesarios para facilitar la higiene personal; Proporciona privacidad durante el uso del baño, el cambio de pañales o el baño; Proporciona apoyo y/o materiales necesarios para facilitar el vestirse; Explica a la persona mayor cuál es la manera correcta de transferirse de lugar; Proporciona apoyo y/o materiales necesarios para que la persona mayor se transfiera de lugar; Proporciona apoyo y/o materiales necesarios para posicionar a la persona mayor.

Solo en el GI hubo un aumento estadísticamente significativo en las siguientes cuestiones: Controla la ingesta de alimentos; Ayuda en la administración de medicamentos según la prescripción médica; Ayuda a la persona a transferirse de lugar; Evalúa la necesidad de cambiar la posición del cuerpo de la persona mayor. Por otro lado, hubo una reducción estadísticamente significativa en el ítem: Mantiene una apariencia bien cuidada, en este grupo.

Hubo un aumento estadísticamente significativo solo en el GC en los ítems: Introduce agua si la sonda se obstruye durante la administración de la dieta y medicamentos; Introduce agua para lavar la sonda después de la administración de la dieta y medicamentos; Proporciona apoyo y/o materiales necesarios para facilitar las eliminaciones urinarias e intestinales; Ayuda con la higiene íntima después de usar el baño o cambiar los pañales; Evalúa la capacidad de la persona mayor para transferirse de lugar.

Se observa una mejora similar en la capacitación de los cuidadores de ambos grupos. No se observaron cambios estadísticamente significativos en las puntuaciones de capacitación en los demás ítems.

**Table:** Tabla 3 Efectos de la intervención en los puntajes de la ECCIAD-ACV* de los cuidadores familiares (n = 58). Porto Alegre, RS, Brasil, 2024

Ítems	**GI** ^†^ **Media ± DE** ^||^	**GC** ^‡^ **Media ± DE** ^||^	**p**_ajustado_^§^
Total			
Basal	2,34 ± 0,44	2,34 ± 0,41	0,812
Final	2,66 ± 0,31	2,72 ± 0,19	0,269
Diferencia	0,31 (0,15 a 0,48)	0,38 (0,19 a 0,57)	0,604
p	<0,001	<0,001	
Prepara las comidas de acuerdo con la dieta prescrita u orientada.			
Basal	2,45 ± 0,87	2,69 ± 0,60	0,065
Final	2,68 ± 0,65	2,82 ± 0,39	0,225
Diferencia	0,23 (-0,11 a 0,58)	0,13 (-0,10 a 0,36)	0,405
p	0,187	0,268	
Prepara la comida de manera adecuada.			
Basal	2,48 ± 0,79	2,62 ± 0,56	0,365
Final	2,74 ± 0,54	2,86 ± 0,47	0,581
Diferencia	0,26 (-0,12 a 0,63)	0,24 (-0,06 a 0,54)	0,944
p	0,181	0,112	
Coloca los alimentos y cubiertos en el lado en que la persona mayor presenta mayor dependencia para estimular el miembro afectado.			
Basal	1,61 ± 1,32	1,41 ± 1,09	0,913
Final	2,24 ± 1,09	2,14 ± 1,24	0,781
Diferencia	0,63 (0,02 a 1,24)	0,73 (0,23 a 1,23)	0,766
p	0,042	0,004	
Proporciona apoyo y/o materiales necesarios para facilitar la alimentación.			
Basal	2,56 ± 0,64	2,57 ± 0,63	0,906
Final	2,96 ± 0,20	2,96 ± 0,20	0,979
Diferencia	0,40 (0,17 a 0,64)	0,39 (0,15 a 0,63)	0,913
p	<0,001	0,002	
Controla la ingesta de alimentos.			
Basal	2,41 ± 1,02	2,59 ± 0,84	0,327
Final	2,87 ± 0,34	2,90 ± 0,31	0,366
Diferencia	0,46 (0,09 a 0,83)	0,31 (-0,03 a 0,64)	0,547
p	0,016	0,074	
Vigila la deglución.			
Basal	2,50 ± 1,00	2,63 ± 0,74	0,444
Final	2,79 ± 0,42	2,89 ± 0,32	0,176
Diferencia	0,29 (-0,09 a 0,67)	0,27 (-0,06 a 0,59)	0,906
p	0,132	0,110	
Ayuda en la administración de los medicamentos según la prescripción médica.			
Basal	2,66 ± 0,72	2,79 ± 0,49	0,537
Final	3,00 ± 0,00	2,78 ± 0,42	0,006
Diferencia	0,34 (0,09 a 0,60)	-0,01 (-0,28 a 0,26)	0,074
p	0,009	0,940	
Introduce agua si la sonda se obstruye durante la administración de la dieta y medicamentos.			
Basal	0,60 ± 0,89	0,20 ± 0,45	0,252
Final	2,00 ± 1,73	3,00 ± 0,00	0,364
Diferencia	1,40 (-0,23 a 3,03)	2,80 (2,45 a 3,15)	0,200
p	0,091	<0,001	
Introduce agua para lavar la sonda después de la administración de la dieta y medicamentos.			
Basal	0,60 ± 0,89	0,20 ± 0,45	0,252
Final	2,00 ± 1,73	3,00 ± 0,00	0,364
Diferencia	1,40 (-0,23 a 3,03)	2,80 (2,45 a 3,15)	0,200
p	0,091	<0,001	
Realiza la hidratación de la piel.			
Basal	2,39 ± 0,96	2,39 ± 0,92	0,783
Final	2,41 ± 0,87	2,65 ± 0,70	0,288
Diferencia	0,02 (-0,56 a 0,60)	0,25 (-0,11 a 0,62)	0,507
p	0,949	0,176	
Prepara el material de higiene.			
Basal	2,72 ± 0,53	2,61 ± 0,74	0,646
Final	2,61 ± 0,85	2,86 ± 0,35	0,202
Diferencia	-0,11 (-0,52 a 0,29)	0,26 (-0,07 a 0,58)	0,164
p	0,586	0,118	
Proporciona apoyo y/o materiales necesarios para facilitar la higiene personal.			
Basal	2,72 ± 0,53	2,45 ± 0,91	0,110
Final	3,00 ± 0,00	2,96 ± 0,21	0,177
Diferencia	0,28 (0,09 a 0,46)	0,51 (0,16 a 0,85)	0,222
p	0,004	0,004	
Ayuda en el baño.			
Basal	2,52 ± 0,74	2,41 ± 0,80	0,463
Final	2,50 ± 0,91	2,68 ± 0,48	0,626
Diferencia	-0,02 (-0,59 a 0,56)	0,28 (-0,09 a 0,65)	0,417
p	0,953	0,141	
Ayuda en la higiene oral.			
Basal	2,55 ± 0,78	2,69 ± 0,55	0,676
Final	1,86 ± 1,35	2,86 ± 0,36	0,065
Diferencia	-0,69 (-1,67 a 0,28)	0,16 (-0,14 a 0,47)	0,168
p	0,162	0,296	
Mantiene una apariencia bien cuidada.			
Basal	2,93 ± 0,26	2,69 ± 0,71	0,048
Final	2,67 ± 0,58	2,75 ± 0,55	0,743
Diferencia	-0,26 (-0,51 a -0,01)	0,06 (-0,31 a 0,43)	0,164
p	0,038	0,752	
Proporciona privacidad durante el uso del baño, el cambio de pañales o el baño.			
Basal	2,46 ± 0,84	2,54 ± 0,79	0,532
Final	2,82 ± 0,73	2,88 ± 0,33	0,546
Diferencia	0,36 (0,06 a 0,66)	0,35 (0,01 a 0,69)	0,966
p	0,018	0,046	
Proporciona apoyo y/o materiales necesarios para facilitar las eliminaciones urinarias e intestinales.			
Basal	2,48 ± 0,80	2,30 ± 0,72	0,473
Final	2,79 ± 0,71	2,95 ± 0,22	0,337
Diferencia	0,31 (-0,10 a 0,71)	0,65 (0,35 a 0,95)	0,199
p	0,138	<0,001	
Ayuda con la higiene íntima después de usar el baño o cambiar pañales.			
Basal	2,22 ± 0,97	2,20 ± 0,91	0,751
Final	2,25 ± 1,17	2,85 ± 0,38	0,395
Diferencia	0,03 (-0,74 a 0,79)	0,65 (0,25 a 1,05)	0,297
p	0,943	0,002	
Proporciona apoyo y/o materiales necesarios para facilitar el vestirse.			
Basal	2,57 ± 0,69	2,69 ± 0,54	0,561
Final	3,00 ± 0,00	2,91 ± 0,29	0,178
Diferencia	0,43 (0,18 a 0,68)	0,22 (0,01 a 0,43)	0,254
p	<0,001	0,037	
Ayuda a la persona a vestirse.			
Basal	2,46 ± 0,79	2,70 ± 0,54	0,153
Final	2,72 ± 0,46	2,71 ± 0,46	0,811
Diferencia	0,26 (-0,06 a 0,58)	0,01 (-0,28 a 0,30)	0,289
p	0,115	0,942	
Evalúa la capacidad de la persona mayor para transferirse de lugar.			
Basal	2,28 ± 0,99	2,36 ± 0,95	0,724
Final	2,67 ± 0,70	2,82 ± 0,50	0,311
Diferencia	0,39 (-0,07 a 0,85)	0,46 (0,06 a 0,86)	0,774
p	0,095	0,025	
Explica a la persona mayor cuál es la manera correcta de transferirse de lugar.			
Basal	2,24 ± 0,91	2,00 ± 1,07	0,639
Final	2,71 ± 0,69	2,65 ± 0,57	0,849
Diferencia	0,47 (0,09 a 0,84)	0,65 (0,21 a 1,10)	0,594
p	0,015	0,004	
Proporciona apoyo y/o materiales necesarios para que la persona mayor se transfiera de lugar			
Basal	2,17 ± 0,97	2,03 ± 0,94	0,729
Final	2,86 ± 0,64	2,95 ± 0,22	0,403
Diferencia	0,69 (0,37 a 1,02)	0,92 (0,55 a 1,28)	0,344
p	<0,001	<0,001	
Ayuda a la persona mayor a transferirse de lugar.			
Basal	2,21 ± 0,98	2,39 ± 0,74	0,268
Final	2,67 ± 0,73	2,76 ± 0,56	0,504
Diferencia	0,46 (0,10 a 0,82)	0,37 (-0,05 a 0,80)	0,658
p	0,011	0,085	
Utiliza una postura adecuada para transferir a la persona mayor de lugar.			
Basal	1,61 ± 1,07	1,68 ± 0,98	0,481
Final	1,95 ± 1,00	2,00 ± 0,86	0,659
Diferencia	0,35 (-0,11 a 0,81)	0,32 (-0,11 a 0,75)	0,766
p	0,138	0,142	
Proporciona apoyo y/o materiales necesarios para posicionar a la persona mayor.			
Basal	2,15 ± 0,91	2,36 ± 0,91	0,398
Final	2,94 ± 0,24	2,80 ± 0,52	0,300
Diferencia	0,80 (0,48 a 1,12)	0,44 (0,02 a 0,86)	0,222
p	<0,001	0,040	
Evalúa la necesidad de cambiar la posición del cuerpo de la persona mayor.			
Basal	2,29 ± 0,90	2,31 ± 0,85	0,961
Final	2,65 ± 0,61	2,43 ± 0,65	0,275
Diferencia	0,36 (0,05 a 0,67)	0,12 (-0,32 a 0,55)	0,353
p	0,022	0,593	
Utiliza una postura adecuada para posicionar correctamente cada parte del cuerpo de la persona mayor.			
Basal	1,74 ± 1,13	1,61 ± 1,07	0,646
Final	2,17 ± 0,79	1,95 ± 0,83	0,395
Diferencia	0,43 (-0,12 a 0,98)	0,34 (-0,11 a 0,80)	0,898
p	0,129	0,138	
Cambia la posición del cuerpo de la persona mayor cuando está acostada.			
Basal	2,16 ± 1,07	2,13 ± 0,95	0,901
Final	2,57 ± 0,79	2,11 ± 0,93	0,251
Diferencia	0,41 (-0,31 a 1,13)	-0,01 (-0,75 a 0,72)	0,417
p	0,262	0,970	

*ECCIAD–AVC = Escala de Capacidades del Cuidador Informal de Ancianos Dependientes por Accidente Cerebrovascular; ^†^GI = Grupo intervención; ^‡^GC = Grupo control; ^§^Efecto de la intervención entre los grupos según el modelo de Ecuaciones de Estimaciones Generalizadas (GEE) complementado por la prueba *Least Significant Difference* (LSD), con valor de p ajustado para el parentesco; ^¶^Media ± desviación estándar

El efecto de la intervención sobre la sobrecarga del cuidador familiar se presenta en la Tabla 4. No hubo diferencias significativas entre los grupos. En cuanto a la sobrecarga total, se observó una reducción estadísticamente significativa en ambos grupos (p<0,001). En las comparaciones de los dominios intragrupo, hubo una reducción estadísticamente significativa en los dominios de tensión general, aislamiento y entorno en ambos grupos. En el dominio de decepción, se observó una reducción significativa solo en el GI (p=0,011).

**Table:** Tabla 4 Efectos de la intervención en los puntajes de la CBS* de los cuidadores familiares (n = 58). Porto Alegre, RS, Brasil, 2024

**Dominios**	**GI** ^†^ **Media ± DE** ^||^	**GC** ^‡^ **Media ± DE** ^||^	**p**_ajustado_^§^
CBS* total			
Basal	1,87 ± 0,46	2,01 ± 0,51	0,570
Final	1,53 ± 0,43	1,60 ± 0,51	0,912
Diferencia	-0,34 (-0,52 a -0,17)	-0,41 (-0,64 a -0,18)	0,679
p	<0,001	<0,001	
Tensión general			
Basal	2,02 ± 0,65	2,12 ± 0,76	0,916
Final	1,69 ± 0,68	1,64 ± 0,69	0,533
Diferencia	-0,33 (-0,64 a -0,03)	-0,48 (-0,82 a -0,14)	0,542
p	0,033	0,006	
Aislamiento			
Basal	1,67 ± 0,62	2,06 ± 1,01	0,099
Final	1,24 ± 0,43	1,46 ± 0,74	0,296
Diferencia	-0,43 (-0,71 a -0,15)	-0,60 (-0,94 a -0,26)	0,443
p	0,003	<0,001	
Decepción			
Basal	1,90 ± 0,75	1,83 ± 0,62	0,417
Final	1,54 ± 0,47	1,57 ± 0,61	0,841
Diferencia	-0,36 (-0,64 a -0,08)	-0,26 (-0,54 a 0,02)	0,555
p	0,011	0,069	
Involucramiento emocional			
Basal	1,14 ± 0,24	1,27 ± 0,63	0,462
Final	1,20 ± 0,38	1,16 ± 0,36	0,467
Diferencia	0,06 (-0,04 a 0,16)	-0,11 (-0,37 a 0,15)	0,227
p	0,254	0,392	
Entorno			
Basal	2,26 ± 0,63	2,50 ± 0,79	0,409
Final	1,70 ± 0,57	1,94 ± 0,72	0,350
Diferencia	-0,56 (-0,84 a -0,28)	-0,56 (-0,90 a -0,21)	0,974
p	<0,001	0,001	

*CBS = *Caregiver Burden Scale*; ^†^GI = Grupo intervención; ^‡^GC = Grupo control; ^§^Efecto de la intervención entre los grupos según el modelo de Ecuaciones de Estimaciones Generalizadas (GEE) complementado por la prueba *Least Significant Difference* (LSD), con valor de p ajustado para el parentesco; ^||^Media ± desviación estándar

En cuanto a los datos de acceso al curso, el promedio total de accesos fue de 10,3 (± 5,4) y el promedio del número de módulos accedidos fue de 7,8 (± 2,5). No hubo interferencia estadísticamente significativa del total de accesos (p=0,164) ni del número de módulos accedidos (p=0,182) en el cambio de los puntajes de la ECCIAD-ACV en el GI. El módulo más accedido fue el Módulo 1 - ¿Qué es un ACV? (36 accesos), seguido por el Módulo 4 - Cuidados con medicamentos (32 accesos). El menos accedido fue el Módulo 11 - Cuidados con traqueostomía (14 accesos).

## Discusión

Este EPR analizó los efectos de una intervención educativa virtual dirigida a cuidadores familiares de personas mayores sobrevivientes de ACV. Se observó una similitud entre los dos grupos en cuanto a la mejora en la capacitación y la reducción de la sobrecarga de los cuidadores. El principal hallazgo de este estudio es que la intervención favoreció significativamente a los cuidadores que la recibieron, mejorando su capacidad para la administración de medicamentos.

Con respecto a la caracterización de los participantes, la mayoría de los cuidadores eran mujeres, con una edad promedio de alrededor de 50 años y una mayor proporción de hijos en el GC en comparación con el GI. Este dato llama la atención, ya que en Brasil, el rol de cuidar a la persona mayor dependiente ha sido históricamente asignado a las hijas y a las esposas/compañeras^(24)^. Este factor podría haber influido en los resultados obtenidos, dado que los hijos asumen la responsabilidad del cuidado por sentirse obligados, pero ese sentimiento de deber puede transformarse en motivación e inspiración para cuidar a sus padres mayores, a pesar de las dificultades para adaptarse a este nuevo rol^(25)^.

Además, en cuanto a las características de los participantes, se observó que en el GC había más cuidadores que recibieron capacitación formal para cuidar antes del estudio. Además, en este grupo, el 75,9% de los participantes ya había cuidado a otra persona dependiente, y la misma situación se observó en el 69% de los cuidadores del GI. Estos datos sugieren que, posiblemente, los cuidadores ya habían desarrollado cierta autoconfianza sobre su competencia, conocimientos y habilidades para realizar tareas diarias y manejar sus emociones^(26)^, lo que podría haber reducido la influencia de la intervención.

En cuanto a las características de las personas mayores, las puntuaciones promedio de la escala MIF indicaron que los sobrevivientes de ACV eran menos dependientes en la evaluación final en ambos grupos, aunque no hubo significancia estadística. Esto podría explicarse considerando que la mayoría sufrió un evento isquémico, que tiende a generar menor dependencia a lo largo del tiempo en comparación con el hemorrágico^(27)^.

Al analizar cada ítem de la escala ECCIAD-ACV, se observa una mejora en cuatro ítems en el GI y en cinco ítems en el GC, lo que sugiere un incremento similar en la capacitación de los cuidadores, que podría atribuirse a la experiencia misma del cuidado. En el ítem sobre la administración de medicamentos, los cuidadores del GI mostraron mejores resultados al final de la intervención, aunque el cambio entre los grupos no fue significativo. El módulo sobre cuidados con medicamentos fue el más accedido por los cuidadores, siendo superado solo por el módulo introductorio sobre qué es el ACV. Un estudio con cuidadores familiares de personas mayores dependientes identificó que el cuidado con los medicamentos era una de las actividades más difíciles de realizar debido a la falta de información y conocimiento^(28)^.

El estudio portugués que desarrolló el instrumento original *Escala de Capacidades do Prestador Informal de Cuidados de Idosos Dependentes por AVC* (ECPICID-AVC), validado posteriormente para su uso en Brasil como ECCIID-AVC^(19)^, aplicó un programa de capacitación llamado *Intervention in Informal Caregivers who take Care of Older People after a Stroke* (InCARE). En este estudio, 85 cuidadores familiares de personas mayores sobrevivientes de ACV recibieron orientación destinada a aumentar sus conocimientos y habilidades prácticas, por medio de visitas domiciliarias (VDs) y llamadas telefónicas durante tres meses tras el alta. El grupo de control (n = 89) recibió el cuidado habitual de salud. Los resultados indicaron que el grupo experimental obtuvo una mayor puntuación promedio en la ECPICAD-ACV a lo largo del tiempo y una reducción de la sobrecarga^(29)^. Hasta el momento, no se han encontrado otros estudios que hayan utilizado la ECCIAD-ACV para evaluar la capacitación de los cuidadores después de una intervención.

No obstante, la literatura identifica diversos estudios de intervención enfocados en mejorar la capacitación de cuidadores familiares de personas dependientes por ACV. Un ensayo clínico aleatorizado (ECA) chino evaluó los efectos de una intervención psicoeducativa centrada en la familia (*Family-focused dyadic psychoeducational intervention* – FDPEI) sobre la sobrecarga y la competencia del cuidador. La fase pre-alta incluyó tres sesiones presenciales y se centró en la preparación para la transición del hospital al hogar; la fase post-alta consistió en cuatro llamadas telefónicas semanales de asesoramiento, incentivando a los cuidadores a identificar dificultades y motivándolos a enfrentarlas. Se observó una reducción significativa de la sobrecarga y una mejora en la competencia del cuidador para brindar cuidados^(30)^.

Un estudio tailandés también ofreció una intervención realizada por enfermeros, que consistía en sesiones diseñadas para aumentar las habilidades conductuales de los cuidadores basadas en información y motivación. Con una duración de ocho semanas, la intervención incluyó una fase pre-alta con orientaciones sobre el ACV y 2 visitas domiciliarias (VDs) post-alta. La intervención mejoró las habilidades de cuidado de los cuidadores familiares^(31)^.

A diferencia de la intervención *e-share*, el programa FDPEI y el estudio tailandés realizaron múltiples sesiones presenciales pre-alta. Se sabe que para que el cuidado domiciliario satisfaga las necesidades de la persona mayor con ACV y reduzca los impactos sobre el cuidador, debe prepararse y planificarse antes del alta hospitalaria^(8)^. La realización de solo una sesión presencial antes del alta puede no haber sido suficiente para obtener mejores resultados en este estudio.

Un estudio realizado en Hong Kong aplicó una intervención realizada por un equipo multidisciplinario mediante un programa educativo de 26 semanas. El grupo de intervención mostró mejoras significativas en términos de competencia de cuidado y un menor nivel de sobrecarga tres meses después de la intervención^(32)^. Un estudio realizado en Perú evaluó el efecto de ocho videos educativos sobre posicionamiento, movilización y transferencias, elaborados por un equipo multiprofesional, para mejorar las habilidades prácticas y el conocimiento de diez cuidadores informales de pacientes con ACV. Las puntuaciones de habilidades prácticas y conocimiento aumentaron significativamente^(33)^.

Las investigaciones realizadas en Hong Kong y Perú ofrecieron una combinación de estrategias de cuidado mediante un equipo multidisciplinario, lo que podría explicar la ausencia de diferencias en los puntajes de capacitación y sobrecarga de los cuidadores en el estudio *e-share*. Sin embargo, en el estudio realizado en Perú, destaca la limitación de que los investigadores no evaluaron a los cuidadores después de la experiencia de cuidado y la muestra fue reducida.

En cuanto a la sobrecarga enfrentada por los cuidadores familiares de personas mayores sobrevivientes de ACV, está bien documentada en la literatura; especialmente en lo que respecta a la transición al hogar^(34)^. Se espera que los cuidadores mejor capacitados experimenten una menor sobrecarga. Un estudio nigeriano evaluó el papel moderador de la preparación para el cuidado en la sobrecarga de los cuidadores familiares. Los resultados mostraron que la preparación moderaba la relación entre sobrecarga y la salud física y mental de los cuidadores^(35)^.

Garantizar que los cuidadores familiares estén adecuadamente preparados y reciban apoyo puede reducir los impactos negativos del cuidado^(35)^. Entre los cuidadores del estudio *e-share*, se observó una reducción de la sobrecarga total en ambos grupos. A pesar de no haber un efecto significativo de la intervención entre los grupos, hubo una reducción significativa en el dominio “Decepción” solo en el GI, lo que puede explicarse por el hecho de que la intervención ofreció disponibilidad de contacto, reforzó la importancia del involucramiento de todos los miembros de la familia en el cuidado y subrayó la necesidad de que el cuidador principal tuviera momentos de descanso, información contenida en el MOOC en el Módulo 2.

A pesar de no haberse observado una interferencia significativa entre el acceso al MOOC y el cambio en los puntajes de la ECCIAD-ACV, se percibe coherencia entre los módulos más accedidos y las necesidades de cuidado identificadas.

Un estudio piloto de ensayo clínico aleatorizado (ECA**)** realizado en los Estados Unidos evaluó la viabilidad y aceptabilidad de una intervención de soporte y solución de problemas por medio de internet y teléfono para cuidadores de sobrevivientes de ACV. La intervención, de dos brazos y administrada por enfermeros, utilizó como base el sitio *web* RESCUE, que incluye las secciones: (1) fichas informativas, (2) lista de recursos adicionales, (3) autogestión, (4) glosario de términos relacionados con el ACV, (5) testimonios, (6) módulo de capacitación y (7) diario de resolución de problemas. La intervención se realizó por teléfono en cuatro u ocho sesiones semanales de 30-60 minutos cada una, adaptadas a los problemas de cada cuidador, durante ocho semanas después del alta hospitalaria. Los resultados indicaron respuestas positivas a la intervención, y el análisis cualitativo reveló que la intervención fue valorada y aceptada por los cuidadores^(36)^.

Es notable que el estudio mencionado, con efectos significativos, fue desarrollado en un país con condiciones sociales y económicas más favorables, lo que difiere de las características encontradas en Brasil. Aun así, se destaca que el uso de estas modalidades contribuye a la universalización del acceso digital y de la información en salud.

En los últimos 15 años, las intervenciones educativas para cuidadores se han realizado en entornos reales, y con la pandemia del coronavirus de 2019, las investigaciones en entornos virtuales se intensificaron. Una revisión sistemática con metaanálisis buscó elucidar las evidencias relacionadas con el uso de *e-Health* (herramientas y soluciones digitales y/o virtuales destinadas a la salud) en la mejora de la calidad de vida (CV) de los cuidadores informales de pacientes dependientes por ACV. El análisis concluyó que hubo una tendencia a mejorar la salud mental de los cuidadores, una mejor capacidad para resolver problemas relacionados con el cuidado y la prevención de problemas derivados de la sobrecarga^(10)^.

Este EPR tiene limitaciones. La mayoría de las personas mayores sobrevivientes de ACV fueron identificadas en la UCE-ACV (23 GI vs 19 GC), donde los cuidadores también contaban con el apoyo de profesionales de la salud con conocimientos altamente especializados. Este hecho, junto con la característica de la muestra, en la que los participantes de ambos grupos tenían experiencia previa como cuidadores, podría haber contribuido a que se encontraran resultados no significativos. En un estudio futuro, sería necesaria una muestra más inclusiva de personas mayores de instituciones no especializadas.

Se infiere que la evaluación de los resultados en un tiempo de seguimiento mayor también podría influir en los resultados, ya que la propia experiencia del cuidado podría haber favorecido la similitud de resultados entre los grupos. En Brasil, enfermeras condujeron el ECA *Nursing Home Care Intervention Post Stroke* (SHARE), que consistió en preparar a los cuidadores para realizar las AVDs de las personas mayores que sufrieron ACV, después del alta. Los resultados mostraron una CV similar y un aumento en la sobrecarga total en el GC y el GI. No obstante, en lo que respecta a la CV, los cuidadores del GI presentaron puntajes más altos en relaciones sociales y, en cuanto a la sobrecarga, el GI mostró menor aislamiento. La evaluación de los resultados se realizó a los 60 días y un año después del alta hospitalaria^(37 - 38)^.

Además, se destaca la ausencia de investigaciones que permitan comparar los cambios en los puntajes de la ECCIAD-ACV como una limitación. Se espera que este estudio estimule a otros investigadores a adoptar su uso, así como la expansión del uso de tecnologías virtuales en investigaciones brasileñas.

Más allá de la investigación, este estudio tiene aplicaciones prácticas, ya que los datos serán divulgados al servicio de salud y el MOOC estará disponible para los cuidadores del GC y para el público en general en una plataforma abierta. Se espera que, de esta manera, se pueda apoyar a los cuidadores familiares que no tienen acceso a servicios de salud con equipos especializados.

Este es el primer estudio en Brasil que propone el uso del MOOC como herramienta educativa para cuidadores familiares de personas mayores sobrevivientes de ACV, representando un importante avance en la Enfermería en la construcción de tecnologías educativas digitales.

## Conclusión

La intervención multicomponente *e-share*, que incluyó un MOOC, una línea telefónica y cuatro contactos programados, no mostró un efecto significativo en la mejora de la capacitación ni en la reducción de la sobrecarga de los cuidadores. Sin embargo, favoreció de manera significativa a los cuidadores del GI en cuanto a la mejora de la administración de medicamentos y la reducción de la sobrecarga en el dominio de decepción.

Este estudio revela la complejidad de las acciones de transición del cuidado tras un ACV. El uso de tecnologías virtuales sencillas explora alternativas para esta transición que brinden apoyo a los cuidadores en la adquisición de habilidades y conocimientos. Es necesario que los cuidadores sean evaluados antes del alta hospitalaria de la persona mayor, garantizando una transición adecuada y reduciendo los efectos negativos del cuidado, destacando el papel central del enfermero en estas acciones.
